# Supplementary material for: Reliability of a wearable wireless patch for continuous remote monitoring of vital signs in patients recovering from major surgery: a clinical validation study from the TRaCINg trial
Source: BMJ Open. 2019 Aug 15;9(8):e031150. doi: 10.1136/bmjopen-2019-031150 (PMC6701670; doi:10.1136/bmjopen-2019-031150)
Supplement: Supplementary data [file bmjopen-2019-031150supp001.pdf]

Supplementary Materials

Sensitivity analysis

Window size	HR (n = 1135)	RR (n = 1134)	Temp (n = 1132)
±2 mins	306	630	212
±5 mins	249	392	174
±10 mins	232	286	147

Missing pairs of data (i.e. no data within n minute window of nurse observation)

Window size	HR (bias, LoA)	RR (bias, LoA)	Temp (bias, LoA)
±2 mins	- 2.74 (-25.39, 19.91)	3.07 (-9.05 to 15.20)	0.82 (-1.21 to 2.86)
±5 mins	-2.35 (-24.68 to 19.98)	3.13 (-8.64 to 14.90)	0.82 (-1.23 to 2.87)
±10 mins	- 1.85 (-23.92 to 20.22)	2.93 (-8.19 to 14.05)	0.82 (-1.13 to 2.78)

Bland-Altman bias and 95% limits of agreement for ±2, ±5, ±10 window lengths of continuous vital sign patch data

Downey C, *et al. BMJ Open* 2019; 9:e031150. doi: 10.1136/bmjopen-2019-031150
