# Supplementary material for: Reliability of a wearable wireless patch for continuous remote monitoring of vital signs in patients recovering from major surgery: a clinical validation study from the TRaCINg trial
Source: BMJ Open. 2019 Aug 15;9(8):e031150. doi: 10.1136/bmjopen-2019-031150 (PMC6701670; doi:10.1136/bmjopen-2019-031150)
Supplement: Author's manuscript [file bmjopen-2019-031150.draft_revisions.pdf]

BMJ Open

BMJ Open is committed to open peer review. As part of this commitment we make the peer review history of every article we publish publicly available.

When an article is published we post the peer reviewers' comments and the authors' responses online. We also post the versions of the paper that were used during peer review. These are the versions that the peer review comments apply to.

The versions of the paper that follow are the versions that were submitted during the peer review process. They are not the versions of record or the final published versions. They should not be cited or distributed as the published version of this manuscript.

BMJ Open is an open access journal and the full, final, typeset and author-corrected version of record of the manuscript is available on our site with no access controls, subscription charges or pay-per-view fees (http://bmjopen.bmj.com).

If you have any questions on BMJ Open's open peer review process please email info.bmjopen@bmj.com

BMJ Open

Reliability of a wearable wireless patch for continuous remote monitoring of vital signs in patients recovering from major surgery: a clinical validation study

Journal:	*BMJ Open*
Manuscript ID	bmjopen-2019-031150
Article Type:	Research
Date Submitted by the Author:	09-May-2019
Complete List of Authors:	Downey, Candice ; University of Leeds, Leeds Institute of Medical Research at St. James's<br>Ng, Shu; University of Leeds, School of Medicine<br>Jayne, David; University of Leeds, Leeds Institute of Medical Research at St. James's<br>Wong, David; University of Leeds, Leeds Institute of Health Sciences
Keywords:	Vital Signs, continuous, monitoring, validation

SCHOLARONE™
Manuscripts

Reliability of a wearable wireless patch for continuous remote monitoring of vital signs in patients recovering from major surgery: a clinical validation study

CL Downey[1], S Ng[2], DG Jayne[1], DWong[3]

1. Leeds Institute of Medicine at St James's, Clinical Sciences Building,
St. James's University Hospital, University of Leeds, Leeds, LS9 7TF
2. School of Medicine, Worsley Building, University of Leeds, LS2 9JT
3. Leeds Institute of Health Sciences, Worsley Building, University of Leeds, LS2 9JT

Correspondence to:

Miss Candice L Downey

Level 7, Clinical Sciences Building

St James's University Hospital

Leeds

LS9 7TF

Email: c.l.downey@leeds.ac.uk

Tel: 0113 2065281

Originality: This article is an original work, has not been published before, and is not being considered for publication elsewhere in its final form, in either printed or electronic media. It is not based on any previous communication to a society or meeting.

Keywords: Remote monitoring, vital signs, continuous, reliability, validation

Word count:

For peer review only

Abstract

Objective

The objective of this study was to validate the accuracy of a wearable remote vital signs monitor to measure heart rate, respiratory rate and temperature in a post-surgical patient population at high risk of complications.

Design

Manually-recorded vital signs data were paired with vital signs data derived from the remote monitor set in patients participating in a trial of continuous remote vital signs monitoring.

Setting

St James's University Hospital, United Kingdom.

Participants

51 patients who had undergone major elective general surgery.

Interventions

The intervention under investigation was the SensiumVitals® monitoring system. This consist of a wireless patch which is worn on the patient's chest and measures heart rate, respiratory rate and temperature continuously. The reference standard was nurse-measured manually-recorded vital signs as part of the National Early Warning Score.

Primary and secondary outcome measures

The primary outcomes were the 95% limits of agreement between manually-recorded vital signs and wearable vital sign patch recordings of heart rate, respiratory rate and temperature. The secondary outcomes were the average percentage completeness of data for each vital sign.

Results

There were 2,737 pairs of matched data. Heart rate measurements showed good correlation (R^2 = 0.67). There was low correlation for respiratory rate (R^2 = 0.01) and temperature (R^2 = 0.13). The average completeness of data were 72.8% for temperature, 59.2% for heart rate and 34.1% for respiratory rate. Distributions of respiratory rate in manually-recorded measurements were statistically implausible.

Conclusions

The remote continuous monitoring system is capable of reliably measuring heart rate, and correlates well with manually-recorded heart rate. The accuracy of respiratory rate and temperature was outside of acceptable limits. Limitations of the remote monitoring system could potentially be

For peer review only

overcome through better signal processing. Inaccuracies in the manually-recorded data present an opportunity to increase awareness amongst staff about the importance of manual observations.

For peer review only

For peer review only - http://bmjopen.bmj.com/site/about/guidelines.xhtml

Article Summary: Strengths and limitations of this study

- Surgical patients are a population likely to benefit from continuous physiological monitoring.
- A large number of paired data sets were available for comparison.
- The reference standard is a clinically relevant comparison, and is standard of care throughout the UK.
- The accuracy of the reference standard is user-dependent.

For peer review only

For peer review only - http://bmjopen.bmj.com/site/about/guidelines.xhtml

1. Introduction

Physiological monitoring using early warning score systems is effective but limited by its intermittent nature (1). It is hypothesised that continuous vital signs monitoring may allow earlier detection of patient deterioration and thereby improve patient outcomes, but existing evidence is limited (2). A consensus of international experts in safety and healthcare technology concluded that, if technically possible and affordable, all patients who are for active treatment should be continuously monitored (3).

Until recently, continuous vital signs monitoring was limited to critical care areas because it required high staff-to-patient ratios and cumbersome equipment which tethered the patient to the bed-space, thereby inhibiting patient mobility and recovery. When hard-wired monitoring was implemented on a general ward, only 16% of patients remained connected in a 72-hour period (4).

New remote monitoring devices, consisting of wearable sensors and aided by wireless data transmission, allow the patient to ambulate freely whilst enjoying the presumed advantages of extra monitoring. Since 2002, a number of such tools have received the United States Food and Drug Administration (FDA) clearance, indicating that they are safe and effective, but clinical studies are required to demonstrate their utility in the inpatient setting (5,6).

A remote monitoring device with a considerable amount of clinical evidence is the SensiumVitals® patch (Figure 1). Attached to the patient's chest with two ECG electrodes, the device monitors heart rate, respiratory rate and skin temperature continuously. The data are transmitted wirelessly every two minutes to a central monitoring station or a mobile device carried by the patient's nurse. This alerts the healthcare worker when there is deviation from pre-set physiological norms, alerting staff to potential patient deterioration.

The patch records respiratory rate by means of impedance pneumography and heart rate through single-lead ECG activity. Temperature is measured by a temperature-sensitive resistor. Once a physiological signal is fully acquired, it is processed by its associated embedded algorithm running inside the in-built processing unit, which enables the transmission of the resultant values to a nearby intranet hot-spot for onward transmission to the central monitoring system.

The underlying technology incorporated into such devices is well understood, but there is limited evidence for its reliability in the clinical setting. One previous study exists which validated the accuracy of the SensiumVitals® system in 61 hospital patients. The patients were monitored at rest for a maximum of two hours, and the device was tested against a conventional bedside clinical monitor using capnographic respiratory rate (7). This does not reflect the true clinical environment, which challenges such devices to provide monitoring continuity over several days in ambulatory patients.

In this study, we validated the accuracy of the SensiumVitals® system to measure heart rate, respiratory rate and temperature in a post-surgical patient population at high risk for complications. The reference standard were manually-recorded vital signs as part of the National Early Warning Score (NEWS). The objective of this study was to assess whether the wireless patch system is able

For peer review only - http://bmjopen.bmj.com/site/about/guidelines.xhtml

For peer review only

to reliably measure vital signs continuously in the clinical setting, and to determine how well it compares to manually-recorded measurements.

2. Methods

Ethical approval was granted on 10th October 2017 by the Yorkshire & The Humber – Leeds West Research Ethics Committee, ref: 17/YH/0180. Informed consent to participate was obtained from all participants in the study.

2.1 Study design

All participants were enrolled in the TRaCINg study, the protocol for which has been published previously (8). This was a single-centre, feasibility, randomised, controlled, parallel group trial of continuous remote vital signs monitoring for patients who had undergone major elective general surgery at St James's University Hospital, Leeds, United Kingdom. Participants were individually randomised on a 1:1 basis to receive either remote monitoring plus NEWS or monitoring by NEWS alone. This paper describes the data from participants randomised to the remote monitoring arm, who wore the SensiumVitals® patch during their hospital admission.

2.2 Patient and public involvement

Patients and the public were involved in the design of the randomised controlled trial, but were not involved in the design of this validation study.

2.3 Data collection

Vital signs data was collected for each participant from two sources. The SensiumVitals® vital sign data were documented at 2-minute intervals and collected from a hospital desktop computer using data-acquisition software developed by Sensium. These data had been pre-processed to discard signals that were subject to gross electrical or motion artefact (7).

NEWS data were collected at regular intervals, depending on the patients' status and based on the NEWS protocol(9). Typically, vital signs were collected at the bedside by members of the nursing staff who were blinded to the SensiumVitals® vital sign data: pulse rate was measured using the pulse oximeter on a multi-parameter portable vital signs monitor; temperature was measured using a tympanic thermometer; respiratory rate was measured manually. The NEWS scores and their component parts were documented electronically. Researchers collected manually-recorded heart rate, respiratory rate and temperature data from the hospital's electronic patient record. Other vital signs collected by the nursing staff as part of the early warning score, such as oxygen saturations, were not extracted.

2.4 Data processing

The two data sources were linked using NHS number and timestamp and consolidated into a single deidentified spreadsheet. Paired data to a NEWS observation was derived from the SensiumVitals® continuous data set by using the median vital sign value within a ±10-minute window of a manually-recorded observation. The time window was used to account for differences between the nurses'

For peer review only - http://bmjopen.bmj.com/site/about/guidelines.xhtml

manually-documented times and the automatic timestamps from the vital sign patch. The median value within this window was used to eliminate the impact of intermittent sensor noise.

2.5 Outcomes

The primary outcomes were the 95% limits of agreement between manual nurse observations and wearable vital sign patch recordings of Heart Rate (HR), Respiratory Rate (RR) and Temperature (Temp). Following precedent, we defined clinical acceptability to be *max* ±10% for HR and RR (or ±3 breaths per minute or ±5 beats per minute) and 0.5°C for Temp (10,11). The secondary outcome was the average percentage completeness of each vital sign.

2.6 Statistical Analysis

For each vital sign, we first visually inspected the paired vital sign measurements via scatter plots, in addition to the raw time series vital signs from the Sensium patch.

Measurements were then formally compared using Bland-Altman analysis. In this analysis, the mean difference between the SensiumVitals® data and the nurse observations and the 95% Limits of Agreement are calculated. We adjusted for repeated measures from the same subject using a model in which time of measurement is modelled as a random effect. This avoids bias caused by differences in number of measurements per patient.

In secondary analysis, we first assessed the average percentage completeness of the data per patient. The numerator was defined as the number of two-minute periods in which vital sign data were provided by the patch. The denominator was the number of 2-minute periods that span the time during which the patch was transmitting data. These time points were preferred to admission and discharge from ward times because the patch may not have been worn for the patient's entire ward admission.

Analyses were undertaken using MATLAB R2017b (The MathWorks Inc., Massachusetts, USA) and the R Methcomp package (12,13).

3. Results

Fifty one patients were recruited to the intervention arm of the TRaCINg study between October 2017 and April 2018. The median number of manually-recorded observation sets was 19 per patient (range 2 to 73 sets of vital signs measurements). There were 2,737 pairs of matched data available for analysis. Vital sign traces for one participant over the course of their entire hospital stay are shown in Figure 2.

3.1 Temperature

Figure 3 is a scatterplot of temperatures recorded by nurses versus those measured by the SensiumVitals® patch. Histograms for each measurement method are presented alongside the x- and y- axes. There is low correlation between the two measurement methods (R^2 = 0.13). The mean and (standard deviation) of manual temperature and wearable temperature were 37.1℃ (0.5℃) and

For peer review only - http://bmjopen.bmj.com/site/about/guidelines.xhtml

36.4°C (1.0°C). Further inspection of the vital sign time series in Figures 1 and 2 shows multiple clinically implausible fluctuations of up to 2 °C within 2 hours within each time series. The mean percentage completeness of temperature data was 72.8%.

Initial visual inspection was therefore sufficient to show that the patch-derived temperature is not a suitable proxy for core temperature, as measured by tympanic thermometer. The Bland-Altmann bias (Figure 4) was 0.82°C, with 95% limits of agreement -1.13°C to 2.78°C.

3.2 Respiratory Rate

Figure 5 shows the scatterplot of nurse-recorded respiratory rate against the SensiumVitals® patch data. There is no correlation between the two measurements methods (R^2 = 0.01). The mean and standard deviation for manual and wearable respiratory rate were 17.6 (1.58) breaths per minute and 15.0 (5.5) breaths per minute, respectively. The mean percentage completeness of respiratory rate data was 31.4%.

Visual inspection of the histogram for manually-recorded respiratory rate shows a large peak at 18 breaths per minute, and a secondary peak at 16 breaths per minute. This result is unexpected for a natural physiological parameter, which may be expected to vary smoothly over the full range of values. Inspection of the vital sign patch histogram indicates a significant proportion of measurements between 5 and 10 breaths per minute. No manually-recorded respiratory rates were recorded in this range. The Bland-Altmann bias (Figure 6) was 2.93 breaths per minute, with 95% limits of agreement -8.19 to 14.05 breaths per minute.

3.3 Heart Rate

Figure 7 shows the scatterplot of nurse-recorded heart rate against the SensiumVitals® patch. There is reasonable correlation between the two measurements (R^2 = 0.67). The mean and (standard deviation) for manual and wearable heart rates are 81.6 (16.2) beats per minute (bpm) and 84.3 (19.3) bpm, respectively. The mean percentage completeness of heart rate data was 59.2%. In addition, visual inspection of the example vital sign traces show good agreement between the measurements (Figures 1 and 2). The Bland-Altmann bias (Figure 8) was 1.85 bpm, with 95% limits of agreement -23.92 to 20.22 bpm.

4. Discussion

In this 51-patient validation study, temperature, respiratory rate, and heart rate measurements obtained from a wearable vital sign patch were compared with manually-recorded observations by nursing staff. Heart rate measurements showed good correlation between the two types of monitoring. There was low correlation between the two measurement methods for respiratory rate and temperature.

For peer review only - http://bmjopen.bmj.com/site/about/guidelines.xhtml

An advantage of the study design is the collection of a large number of data points for analysis. The approach is clinically valid, as the NEWS system is the national standard for vital signs monitoring in the United Kingdom. The surgical patient population is a clinically relevant cohort. There are high rates of complications after major surgery (14), but many surgical complications, such as sepsis, are attenuated by early detection. By virtue of their suitability for surgery, patients experiencing severe complications are likely to be candidates for full active management and escalation of care. They are therefore a population likely to benefit from continuous physiological monitoring.

There are few clinical evaluations of continuous vital signs monitoring in the literature. Previous validation studies have studied participants who are confined to their bed space by wired monitoring equipment (7,10). This is not a valid approach in the surgical setting, where enhanced recovery programmes mandate early mobilisation after surgery. In this study, patients were allowed to ambulate freely as part of their usual postoperative care.

The findings must be interpreted within the limitations of the study. There were a relatively small number of patients in the study. Data completeness was low, especially for respiratory rate, although the data completeness for heart rate and temperature were similar to previous work (10). The reference standard, whilst clinically relevant, is inherently flawed. Early warning scores such as NEWS are known to be limited by their user-dependent nature. In addition, manually-collected vital signs can be subject to the effects of 'white-coat hypertension'; heart rate, respiratory rate and temperature can be elevated simply by the arousal effect of the nurse interaction.

Deficits in the manually-recorded observations were particularly evident in the analysis of respiratory rate. Analysis of the manually-recorded values alone revealed a statistically unlikely preponderance of 18 breaths per minute, with a secondary peak at 16 breaths per minute. This casts doubt on the reliability of these manual measurements. It has been well described that respiratory rate is often miscalculated or omitted when calculated early warning scores (15,16). It is also recognised that clinical staff detect patient status in advance of manual measurements for an early warning score system 'by using information not currently encoded within it.'

The patch data for respiratory rate is also unlikely to be reliable, as a significant proportion of measurements were between 5 and 10 breaths per minute. This proportion of low values is much greater than those described in previously derived distributions from larger populations (17). There are also rapid fluctuations in respiratory rate which are physiologically implausible and may have been affected by patient movement, speech or coughing.

The manually-recorded temperature measurements showed plausible distributions and are likely to be accurate. The low correlation between the nurse-measured temperatures and the patch data can be explained by the difference in measurement techniques. The patch measures skin temperature which may not accurately reflect the tympanic temperature measured by the nursing staff. Skin temperature is highly dependent on environmental factors such as the ambient temperature, clothing and blankets.

The reliability of the continuous temperature measurement is, however, limited. The time series analysis shows evidence of regular patch disconnection, indicated by rapid drops in temperature followed by increases consistent with conductive heating, or warming back up. These warm-up periods render the raw signals unreliable, although this limitation may be overcome through better signal processing. For instance, Clifton et al. used Bayesian change point analysis to detect step

For peer review only - http://bmjopen.bmj.com/site/about/guidelines.xhtml

changes in temperature across a large study population. A similar approach may be used to determine disconnection on an individual patient basis(18).

Conclusions

The SensiumVitals® monitoring system is capable of reliably measuring heart rate, and correlates well with manually-recorded heart rate. The accuracy of respiratory rate and temperature was outside of acceptable limits. Limitations of the remote monitoring system could potentially be overcome through better signal processing. Inaccuracies in manually-recorded data present an opportunity to increase awareness amongst nursing staff about the importance of manual observations.

For peer review only

Figure legends

Figure 1: The SensiumVitals® monitoring patch. Image reproduced with permission from Sensium, Abingdon, UK.

Figure 2: Vital signs data for a single participant. The grey lines show the minute-by-minute vital sign values from the SensiumVitals® patch. The black markers show the median value of the SensiumVitals® vital signs (evaluated from +-10 mins of the nurse observation time). The red markers show the manually-recorded vital signs. Where there is a wide difference between the red and black markers at a single time point, this indicates disagreement between the two vital signs measurement techniques.

Figure 3: Scatter plot and marginal histogram of paired manual and SensiumVitals® temperature observations.

Figure 4: Bland-Altman plot for temperature with limits of agreement adjusted for repeated measures.

Figure 5: Scatter plot and marginal histogram of paired manual and SensiumVitals® respiratory rate observations; rpm=respirations per minute.

Figure 6: Bland-Altman plot for respiratory rate with limits of agreement adjusted for repeated measures.

Figure 7: Scatter plot and marginal histogram of paired manual and SensiumVitals® heart rate observations; bpm=beats per minute.

Figure 8: Bland-Altman plot for heart rate with limits of agreement adjusted for repeated measures.

For peer review only - http://bmjopen.bmj.com/site/about/guidelines.xhtml

For peer review only

For peer review only - http://bmjopen.bmj.com/site/about/guidelines.xhtml

Funding statement

Candice Downey is in possession of a Doctoral Research Fellowship (DRF-2016-09-037) supported by the National Institute for Health Research. DGJ received funding support through an NIHR Research Professorship. The research is supported by the NIHR infrastructure at Leeds.

The views expressed in this publication are those of the authors and not necessarily those of the NHS, the National Institute for Health Research, Health Education England or the Department of Health.

Competing interests

There are no known conflicts of interest associated with this work and there has been no significant financial support for this work that could have influenced its outcome.

Author contributions

CD, SN and DW were involved in the conception of the work. CD designed the study. DW provided methodological expertise. SN undertook the data collection. DW and CD performed the analysis and interpretation. CD and DW drafted the article. All authors were involved in critical revision of the article and have given final approval of the version to be submitted.

Acknowledgements

The authors gratefully acknowledge the assistance of the Leeds Teaching Hospitals NHS Trust Surgical Research Team, with particular thanks to Mrs. Pauline Walton.

Data sharing statement

Research materials related to this work can be accessed on request from the corresponding author.

For peer review only

References

1. Downey CL, Tahir W, Randell R, Brown JM, Jayne DG. Strengths and limitations of early warning scores: A systematic review and narrative synthesis. Vol. 76, International Journal of Nursing Studies. 2017. p. 106–19.

2. Cardona-Morrell M, Prgomet M, Turner RM, Nicholson M, Hillman K. Effectiveness of continuous or intermittent vital signs monitoring in preventing adverse events on general wards: a systematic review and meta-analysis. Vol. 70, International Journal of Clinical Practice. 2016. p. 806–24.

3. DeVita MA, Smith GB, Adam SK, Adams-Pizarro I, Buist M, Bellomo R, et al. Identifying the hospitalised patient in crisis. A consensus conference on the afferent limb of Rapid Response Systems. Resuscitation. 2010;81(4):375–82.

4. Bonnici T, Tarassenko L, Clifton D, Watkinson P. The digital patient. Clin Med (Northfield Il). 2013;13(3):252–7.

5. Michard F. A sneak peek into digital innovations and wearable sensors for cardiac monitoring. J Clin Monit Comput. 2017 Apr;31(2):253–9.

6. Hofmann B, Welch H. New diagnostic tests: more harm than good. Br Med J. 2017;358:j3314.

7. Hernandez-Silveira M, Ahmed K, Ang S-S, Zandari F, Mehta T, Weir R, et al. Assessment of the feasibility of an ultra-low power, wireless digital patch for the continuous ambulatory monitoring of vital signs. BMJ Open. 2015;5(5).

8. Downey CL, Croft J, Buckley H, Randell R, Brown JM, Jayne DG. Trial of Remote Continuous versus Intermittent NEWS monitoring after major surgery (TRaCINg): protocol for a feasibility randomised controlled trial. Pilot Feasibility Stud. 2018;4(1):112.

9. Royal College of Physicians. National Early Warning Score (NEWS) 2: Standardising the assessment of acute-illness severity in the NHS. Updated report of a working party. 2017.

10. Breteler MJM, Huizinga E, van Loon K, Leenen LPH, Dohmen DAJ, Kalkman CJ, et al. Reliability of wireless monitoring using a wearable patch sensor in high-risk surgical patients at a step-down unit in the Netherlands: a clinical validation study. BMJ Open [Internet]. 2018 Feb 1;8(2). Available from: http://bmjopen.bmj.com/content/8/2/e020162.abstract

11. Niven D, Gaudet J, Laupland K, Mrklas K, Roberts D, Stelfox H. Accuracy of peripheral thermometers for estimating temperature: A systematic review and meta-analysis. Ann Intern Med [Internet]. 2015 Nov 17;163(10):768–77. Available from: http://dx.doi.org/10.7326/M15-1150

12. Carstensen B, Gurrin L, Ekstrom C, Figurski M. MethComp: functions for analysis of agreement in method comparison studies. 2015. p. R package version 1.22.2.

13. R Core Team. R: A language and environment Statistical, statistical computing. 2018. p. R Foundation Computing, Vienna, Austria.

For peer review only - http://bmjopen.bmj.com/site/about/guidelines.xhtml

14. The International Surgical Outcomes Study Group. Global patient outcomes after elective surgery: prospective cohort study in 27 low-, middle-, and high-income countries. Br J Anaesth. 2016;117:601–9.

15. Nwulu U, Westwood D, Edwards D, Kelliher F, Coleman J. Adoption of an electronic observation chart with an integrated early warning scoring system on pilot wards: a descriptive report. Comput Informatics, Nurs. 2012;30(7):371–9.

16. Ludikhuize J, Smorenburg S, de Rooij S, de Jonge E. Identification of deteriorating patients on general wards; measurement of vital parameters and potential effectiveness of the Modified Early Warning Score. J Crit Care. 2012;27(4):424.e7-13.

17. Tarassenko L, Clifton DA, Pinsky MR, Hravnak MT, Woods JR, Watkinson PJ. Centile-based early warning scores derived from statistical distributions of vital signs. Resuscitation [Internet]. 2011;82(8):1013–8. Available from: http://www.sciencedirect.com/science/article/pii/S030095721100195X

18. Clifton DA, Wong D, Clifton L, Wilson S, Way R, Pullinger R, et al. A Large-Scale Clinical Validation of an Integrated Monitoring System in the Emergency Department. IEEE J Biomed Heal Informatics. 2013;17(4):835–42.

For peer review only

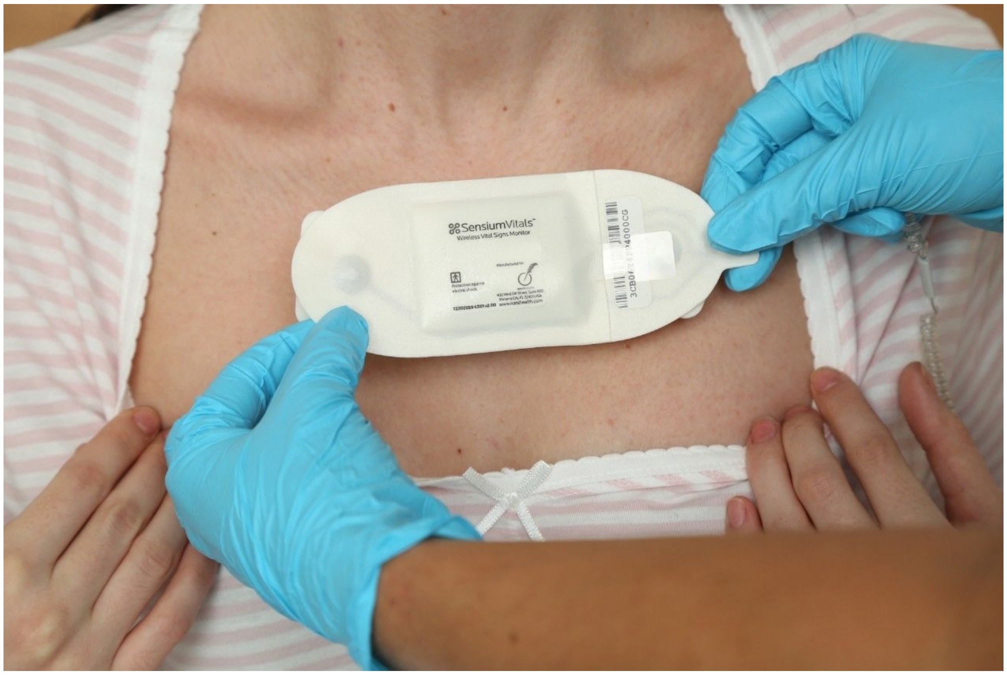

Figure 1: The SensiumVitals® monitoring patch. Image reproduced with permission from Sensium, Abingdon, UK.

451x300mm (72 x 72 DPI)

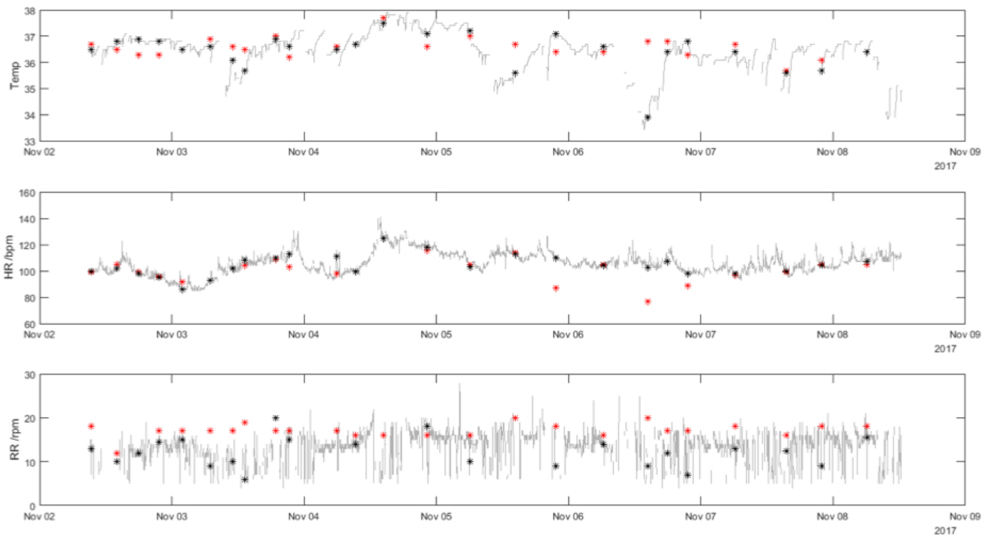

Figure 2: Vital signs data for a single participant. The grey lines show the minute-by-minute vital sign values from the SensiumVitals® patch. The black markers show the median value of the SensiumVitals® vital signs (evaluated from +-10 mins of the nurse observation time). The red markers show the manually-recorded vital signs. Where there is a wide difference between the red and black markers at a single time point, this indicates disagreement between the two vital signs measurement techniques.

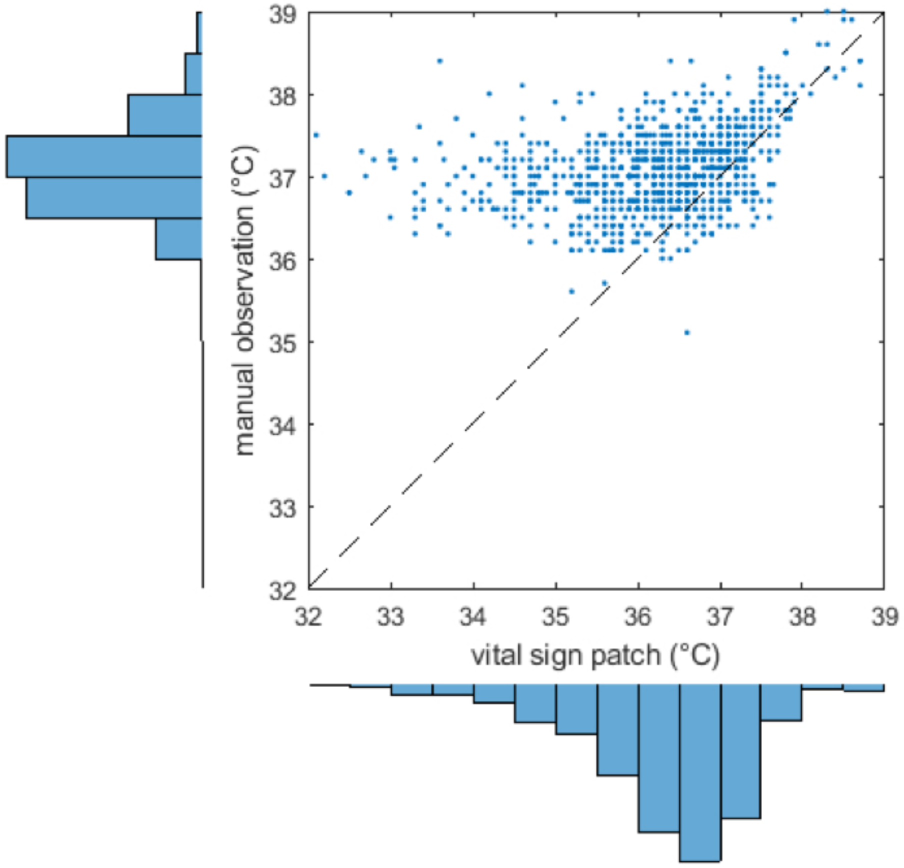

Figure 3: Scatter plot and marginal histogram of paired manual and SensiumVitals® temperature observations.

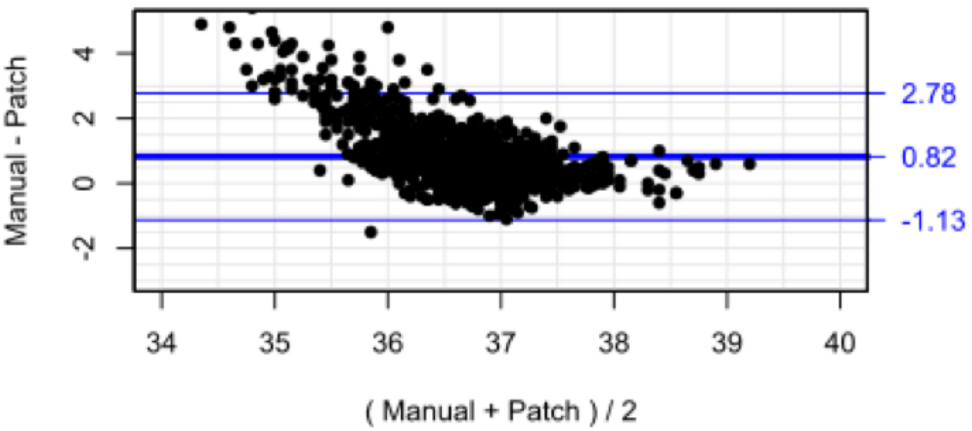

Figure 4: Bland-Altman plot for temperature with limits of agreement adjusted for repeated measures.

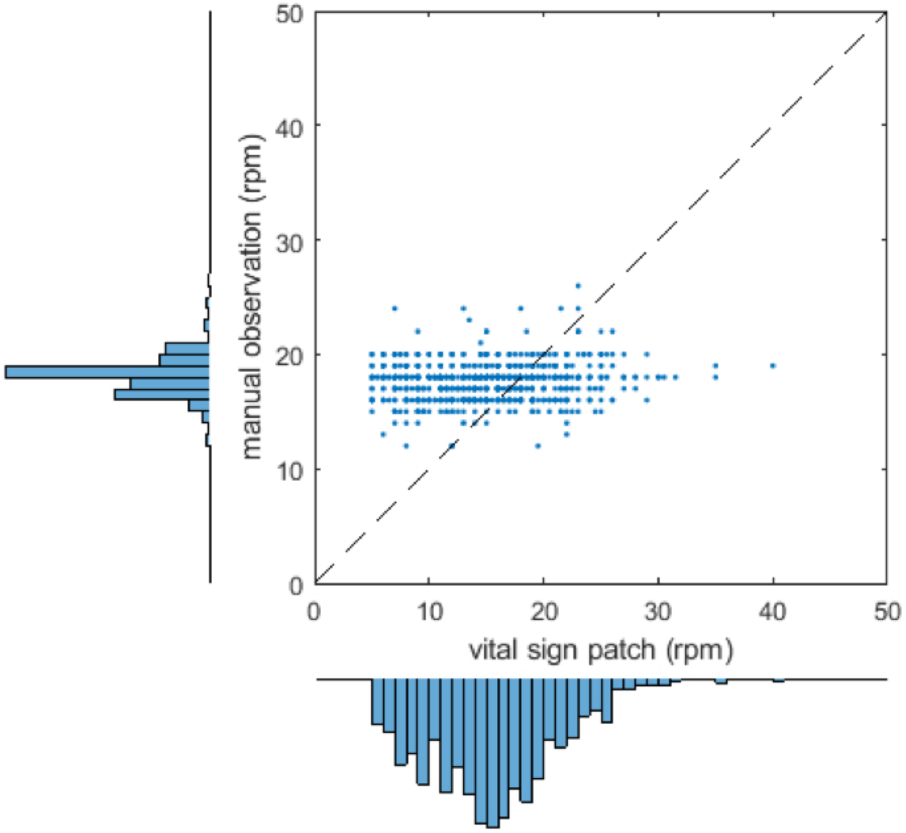

Figure 5: Scatter plot and marginal histogram of paired manual and SensiumVitals® respiratory rate observations; rpm=respirations per minute.

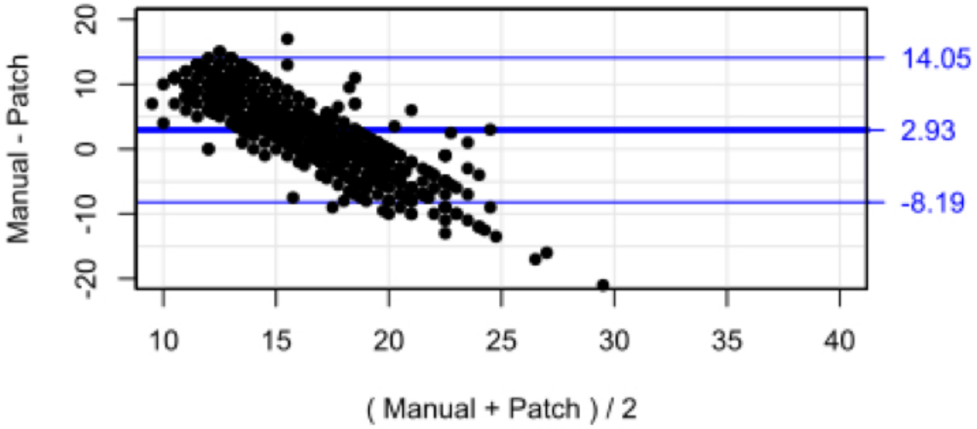

Figure 6: Bland-Altman plot for respiratory rate with limits of agreement adjusted for repeated measures.

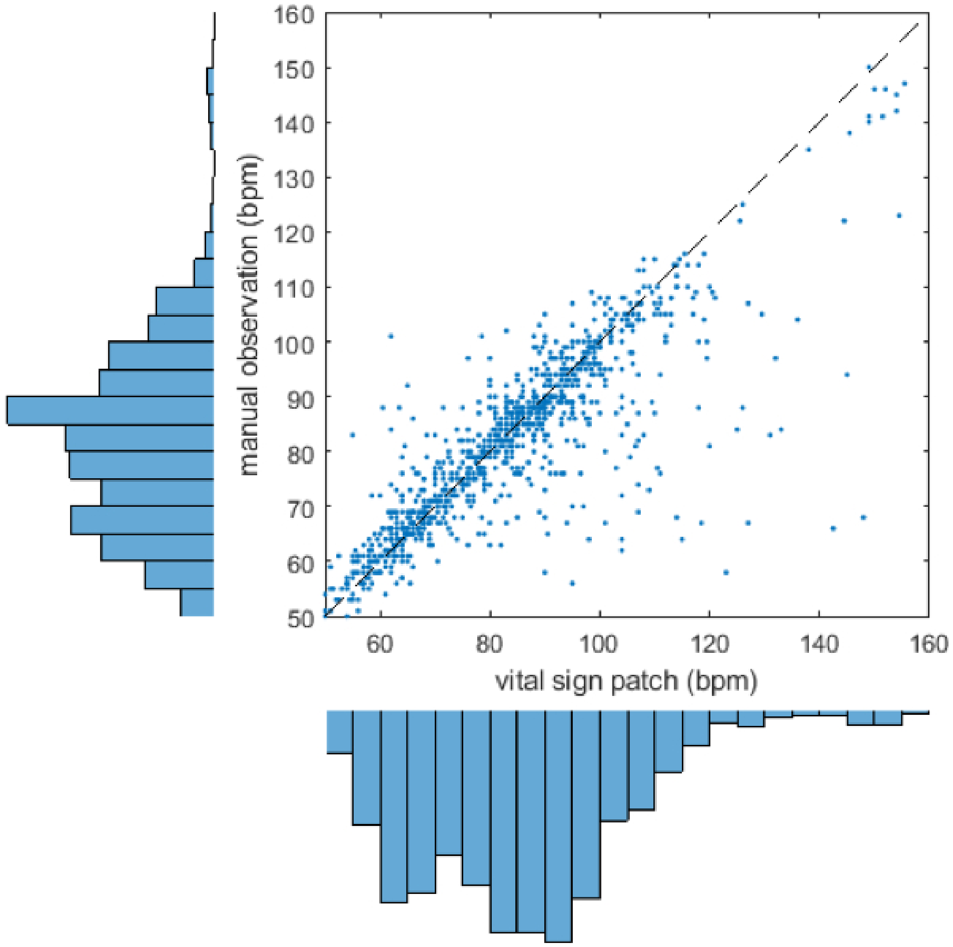

Figure 7: Scatter plot and marginal histogram of paired manual and SensiumVitals® heart rate observations; bpm=beats per minute.

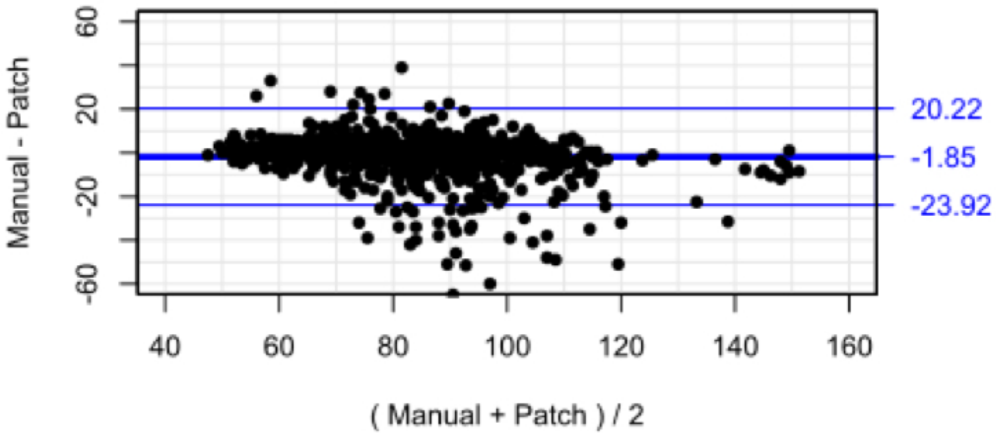

Figure 8: Bland-Altman plot for heart rate with limits of agreement adjusted for repeated measures.

BMJ Open

Reliability of a wearable wireless patch for continuous remote monitoring of vital signs in patients recovering from major surgery: a clinical validation study

Journal:	*BMJ Open*
Manuscript ID	bmjopen-2019-031150.R1
Article Type:	Research
Date Submitted by the Author:	25-Jun-2019
Complete List of Authors:	Downey, Candice ; University of Leeds, Leeds Institute of Medical Research at St. James's<br>Ng, Shu; University of Leeds, School of Medicine<br>Jayne, David; University of Leeds, Leeds Institute of Medical Research at St. James's<br>Wong, David; University of Manchester, Centre for Health Informatics
**Primary Subject Heading**:	Surgery
Secondary Subject Heading:	Evidence based practice, Health informatics
Keywords:	Vital Signs, continuous, monitoring, validation

SCHOLARONE™
Manuscripts

Reliability of a wearable wireless patch for continuous remote monitoring of vital signs in patients recovering from major surgery: a clinical validation study

CL Downey[1], S Ng[2], DG Jayne[1], DWong[3]

1. Leeds Institute of Medicine at St James's, Clinical Sciences Building,

St. James's University Hospital, University of Leeds, Leeds, LS9 7TF

2. School of Medicine, Worsley Building, University of Leeds, LS2 9JT

3. Centre for Health Informatics, University of Manchester, Manchester, M13 9GB

Correspondence to:

Miss Candice L Downey

Level 7, Clinical Sciences Building

St James's University Hospital

Leeds

LS9 7TF

Email: c.l.downey@leeds.ac.uk

Tel: 0113 2065281

For peer review only

Originality: This article is an original work, has not been published before, and is not being considered for publication elsewhere in its final form, in either printed or electronic media. It is not based on any previous communication to a society or meeting.

Keywords: Remote monitoring, vital signs, continuous, reliability, validation

For peer review only - http://bmjopen.bmj.com/site/about/guidelines.xhtml

Abstract

Objective

To validate whether a wearable remote vital signs monitor could accurately measure heart rate, respiratory rate and temperature in a post-surgical patient population at high risk of complications.

Design

Manually-recorded vital signs data were paired with vital signs data derived from the remote monitor set in patients participating in a trial of continuous remote vital signs monitoring.

Setting

St James's University Hospital, United Kingdom.

Participants

51 patients who had undergone major elective general surgery.

Interventions

The intervention was the SensiumVitals® monitoring system. This is a wireless patch worn on the patient's chest that measures heart rate, respiratory rate and temperature continuously. The reference standard was nurse-measured manually-recorded vital signs.

Primary and secondary outcome measures

The primary outcomes were the 95% limits of agreement between manually-recorded and wearable patch vital sign recordings of heart rate, respiratory rate and temperature. The secondary outcomes were the percentage completeness of vital sign patch data for each vital sign.

Results

1,135 nurse observations were available for analysis. There was no clinically meaningful bias in heart rate (1.85 bpm), but precision was poor (95% limits of agreement -23.92 to 20.22 bpm). Agreement was poor for respiratory rate (bias 2.93 breaths per minute, 95% limits of agreement -8.19 to 14.05 breaths per minute) and temperature (bias 0.82°C, 95% limits of agreement -1.13°C to 2.78°C). Vital sign patch data completeness was 72.8% for temperature, 59.2% for heart rate and 34.1% for respiratory rate. Distributions of respiratory rate in manually-recorded measurements were clinically implausible.

Conclusions

The continuous monitoring system did not reliably provide heart rate consistent with nurse measurements. The accuracy of respiratory rate and temperature was outside of acceptable limits. Limitations of the system could potentially be overcome through better signal processing. Whilst

For peer review only - http://bmjopen.bmj.com/site/about/guidelines.xhtml

acknowledging the time pressures placed on nursing staff, inaccuracies in the manually-recorded data present an opportunity to increase awareness about the importance of manual observations, particularly with regard to methods of manual heart rate and respiratory rate measurements.

Article Summary: Strengths and limitations of this study

- Surgical patients are a population likely to benefit from continuous physiological monitoring.
- A large number of paired data sets were available for comparison.
- The reference standard is a clinically relevant comparison, and is standard of care throughout the UK.
- The accuracy of the reference standard is user-dependent.

For peer review only

1. Introduction

Physiological monitoring using early warning score systems is effective but limited by its intermittent nature (1). It is hypothesised that continuous vital signs monitoring may allow earlier detection of patient deterioration and thereby improve patient outcomes, but existing evidence is limited (2). A consensus of international experts in safety and healthcare technology concluded that, if technically possible and affordable, all patients who are for active treatment should be continuously monitored (3).

Until recently, continuous vital signs monitoring was limited to critical care areas because it required high staff-to-patient ratios and cumbersome equipment which tethered the patient to the bed-space, thereby inhibiting patient mobility and recovery. When hard-wired monitoring was implemented on a general ward, only 16% of patients remained connected in a 72-hour period (4).

New remote monitoring devices, consisting of wearable sensors and aided by wireless data transmission, allow the patient to ambulate freely whilst enjoying the presumed advantages of extra monitoring. Since 2002, a number of such tools have received the United States Food and Drug Administration (FDA) clearance, indicating that they are safe and effective, but clinical studies are required to demonstrate their utility in the inpatient setting (5,6).

A remote monitoring device with a considerable amount of clinical evidence is the SensiumVitals® patch (Figure 1)(7–10). Attached to the patient's chest with two ECG electrodes, the device monitors heart rate, respiratory rate and skin temperature continuously. The data are transmitted wirelessly every two minutes to a central monitoring station or a mobile device carried by the patient's nurse. This alerts the healthcare worker when there is deviation from pre-set physiological norms, alerting staff to potential patient deterioration.

The patch records respiratory rate by means of impedance pneumography and heart rate through single-lead ECG activity. Temperature is measured by a temperature-sensitive resistor. Once a physiological signal is fully acquired, it is processed by its associated embedded algorithm running inside the in-built processing unit, which enables the transmission of the resultant values to a nearby intranet hot-spot for onward transmission to the central monitoring system.

The underlying technology incorporated into such devices is well understood, but there is limited evidence for its reliability in the clinical setting. One previous study exists which validated the accuracy of the SensiumVitals® system in 61 hospital patients. The patients were monitored at rest for a maximum of two hours, and the device was tested against a conventional bedside clinical monitor using capnographic respiratory rate (11). This does not reflect the true clinical environment, which challenges such devices to provide monitoring continuity over several days in ambulatory patients.

In this study, we validated the accuracy of the SensiumVitals® system to measure heart rate, respiratory rate and temperature in a post-surgical patient population at high risk for complications. The reference standard were manually-recorded vital signs as part of the National Early Warning Score (NEWS). The objective of this study was to assess whether the wireless patch system is able

For peer review only - http://bmjopen.bmj.com/site/about/guidelines.xhtml

to reliably measure vital signs continuously in the clinical setting, and to determine how well it compares to manually-recorded measurements.

2. Methods

Ethical approval was granted on 10th October 2017 by the Yorkshire & The Humber – Leeds West Research Ethics Committee, ref: 17/YH/0180. Informed consent to participate was obtained from all participants in the study.

2.1 Study design

All participants were enrolled in the TRaCINg study, the protocol for which has been published previously (12). This was a single-centre, feasibility, randomised, controlled, parallel group trial of continuous remote vital signs monitoring for patients who had undergone major elective general surgery at St James's University Hospital, Leeds, United Kingdom. Participants were individually randomised on a 1:1 basis to receive either remote monitoring plus NEWS or monitoring by NEWS alone. This paper describes the data from participants randomised to the remote monitoring arm, who wore the SensiumVitals® patch during their hospital admission. The TRaCINg study is listed on the ISRCTN registry with study ID ISRCTN16601772 (http://www.isrctn.com/ISRCTN16601772).

2.2 Patient and public involvement

Patients and the public were involved in the design of the randomised controlled trial, but were not involved in the design of this validation study.

2.3 Data collection

Vital signs data was collected for each participant from two sources. The SensiumVitals® vital sign data were documented at 2-minute intervals and collected from a hospital desktop computer using data-acquisition software developed by Sensium. These data had been pre-processed to discard signals that were subject to gross electrical or motion artefact (11). Patients were allowed to ambulate whilst wearing the monitoring patch; however, due to the major surgery they had undergone, most patients remained at their bedsides for the duration of their hospital stay.

NEWS data were collected at regular intervals, depending on the patients' status and based on the NEWS protocol(13). Typically, vital signs were collected at the bedside, with the patients either sitting or lying down, by members of the nursing staff who were blinded to the SensiumVitals® vital sign data: pulse rate was measured using the pulse oximeter on a multi-parameter portable vital signs monitor; temperature was measured using a tympanic thermometer; respiratory rate was measured manually. The NEWS scores and their component parts were documented electronically. Researchers collected manually-recorded heart rate, respiratory rate and temperature data from the hospital's electronic patient record. Other vital signs collected by the nursing staff as part of the early warning score, such as oxygen saturations, were not extracted.

2.4 Data processing

The two data sources were linked using NHS number and timestamp and consolidated into a single deidentified spreadsheet. Paired data to a NEWS observation was derived from the SensiumVitals®

For peer review only - http://bmjopen.bmj.com/site/about/guidelines.xhtml

continuous data set by using the median vital sign value within a ±10-minute window of a manually-recorded observation. The time window was used to account for differences between the nurses' manually-documented times and the automatic timestamps from the vital sign patch. The median value within this window was used to eliminate the impact of intermittent sensor noise.

2.5 Outcomes

The primary outcomes were the 95% limits of agreement between manual nurse observations and wearable vital sign patch recordings of Heart Rate (HR), Respiratory Rate (RR) and Temperature (Temp). Following precedent, we defined clinical acceptability to be *max* ±10% for HR and RR (or ±3 breaths per minute or ±5 beats per minute) and 0.5°C for Temp (14,15). The secondary outcome was the average percentage completeness of continuous patch data.

2.6 Statistical Analysis

For each vital sign, we first visually inspected the paired vital sign measurements via scatter plots, in addition to the raw time series vital signs from the Sensium patch.

Measurements were then formally compared using Bland-Altman analysis. In this analysis, the mean difference between the SensiumVitals® data and the nurse observations and the 95% Limits of Agreement are calculated. We adjusted for multiple measurements from the same subject using a model in which time of measurement is modelled as a random effect(16). This avoids bias caused by differences in number of measurements per patient. We also reported the Pearson correlation coefficient and the root mean squared (RMS) error for each vital sign.

In secondary analysis, we first assessed the average percentage completeness of the continuous patch data per patient. The numerator was defined as the number of two-minute periods in which vital sign data were provided by the patch. The denominator was the number of 2-minute periods that span the time during which the patch was transmitting data. These time points were preferred to admission and discharge from ward times because the patch may not have been worn for the patient's entire ward admission. In sensitivity analyses, we repeated both the Bland-Altman analyses using ±2 and ±2 minute windows of continuous data.

Analyses were undertaken using MATLAB R2017b (The MathWorks Inc., Massachusetts, USA) and the R Methcomp package (17,18).

3. Results

Fifty one patients were recruited to the intervention arm of the TRaCINg study between October 2017 and April 2018. The median number of manually-recorded observation sets was 19 per patient (range 2 to 73 sets of vital signs measurements). There were 1,135 nurse observations available for analysis. All observations had a documented heart rate. Four observations had missing observations, 1 for respiratory rate and 3 for temperature. Vital sign traces for one participant over the course of their entire hospital stay are shown in Figure 2.

For peer review only - http://bmjopen.bmj.com/site/about/guidelines.xhtml

3.1 Heart Rate

Figure 3 shows the scatterplot of nurse-recorded heart rate against the SensiumVitals® patch. There is reasonable correlation between the two measurements ($R^2 = 0.67$, $p < 0.001$). The mean and (standard deviation) for manual and wearable heart rates are 81.6 (16.2) beats per minute (bpm) and 84.3 (19.3) bpm, respectively. The mean percentage completeness of continuous patch data for heart rate was 59.2%. In addition, visual inspection of the example vital sign traces show good agreement between the measurements. The Bland-Altman bias (Figure 4) was 1.85 bpm, with 95% limits of agreement -23.92 to 20.22 bpm. The RMS error was 11.25 bpm. The limits of agreement and RMS error exceeded the acceptability criterion.

3.2 Respiratory Rate

Figure 5 shows the scatterplot of nurse-recorded respiratory rate against the SensiumVitals® patch data. There is no correlation between the two measurements methods ($R^2 = 0.01$, $p < 0.001$). The mean and standard deviation for manual and wearable respiratory rate were 17.6 (1.58) breaths per minute and 15.0 (5.5) breaths per minute, respectively. The mean percentage completeness of continuous patch data for respiratory rate data was 31.4%.

Visual inspection of the histogram for manually-recorded respiratory rate shows a large peak at 18 breaths per minute, and a secondary peak at 16 breaths per minute. This result is unexpected for a natural physiological parameter, which may be expected to vary smoothly over the full range of values. Indeed, the peaks do not appear on the vital sign patch histogram. Inspection of the vital sign patch histogram indicates a significant proportion of measurements between 5 and 10 breaths per minute. No manually-recorded respiratory rates were recorded in this range. The Bland-Altman bias (Figure 6) was 2.93 breaths per minute, with 95% limits of agreement -8.19 to 14.05 breaths per minute. The RMS error was 6.14 breaths per minute and the limits of agreement are wider than the pre-specified acceptable error of 3 breaths/min.

3.3 Temperature

Figure 7 is a scatterplot of temperatures recorded by nurses versus those measured by the SensiumVitals® patch. Histograms for each measurement method are presented alongside the x- and y- axes. There is low correlation between the two measurement methods ($R^2 = 0.13$, $p < 0.001$). The mean and (standard deviation) of manual temperature and wearable temperature were 37.1℃ (0.5℃) and 36.4℃ (1.0℃). Further inspection of the vital sign time series in Figures 1 and 2 shows multiple clinically implausible fluctuations of up to 2 °C within 2 hours within each time series. The mean percentage completeness of continuous patch data for temperature was 72.8%.

Initial visual inspection was therefore sufficient to show that the patch-derived temperature is not a suitable proxy for core temperature, as measured by tympanic thermometer. The Bland-Altman bias (Figure 8) was 0.82℃, with 95% limits of agreement -1.13℃ to 2.78℃. The RMS error was 1.28℃. In addition to large systematic bias between the two methods, the limits of agreement did not meet the pre-defined clinical acceptability criterion (0.5°C).

For peer review only - http://bmjopen.bmj.com/site/about/guidelines.xhtml

In a sensitivity analysis, all Bland-Altman analyses was repeated using ±2 and ±5 minute windows of vital sign patch data. There were no meaningful differences in the bias or limits of agreement [Supplementary Material].

4. Discussion

In this 51-patient validation study, temperature, respiratory rate, and heart rate measurements obtained from a wearable vital sign patch were compared with manually-recorded observations by nursing staff. Whilst there was reasonable correlation between the two methods for heart rate measurements (R^2 = 0.67) there were large discrepancies in many instances, as indicated by the Bland Altman analysis (bias 1.85 bpm, 95% limits of agreement -23.92 to 20.22 bpm). It is not clear whether there were errors in the manual observation, in the vital sign patch, or both.

There was low correlation for respiratory rate (R^2 = 0.01)(Bland-Altman bias 2.93 breaths per minute, 95% limits of agreement -8.19 to 14.05 breaths per minute) and temperature (R^2 = 0.13)(Bland-Altman bias 0.82℃, 95% limits of agreement -1.13℃ to 2.78℃). The differences between manual and vital sign patch measurements for all three measured vital signs were outside of acceptable limits. The average completeness of data were 72.8% for temperature, 59.2% for heart rate and 34.1% for respiratory rate.

An advantage of the study design is the collection of a large number of data points for analysis. The approach is clinically valid, as the NEWS system is the national standard for vital signs monitoring in the United Kingdom. The surgical patient population is a clinically relevant cohort. There are high rates of complications after major surgery (19), but many surgical complications, such as sepsis, are attenuated by early detection. By virtue of their suitability for surgery, patients experiencing severe complications are likely to be candidates for full active management and escalation of care. They are therefore a population likely to benefit from reliable continuous physiological monitoring.

There are few clinical evaluations of continuous vital signs monitoring in the literature. Previous validation studies have studied participants who are confined to their bed space by wired monitoring equipment (11,14). In the surgical setting, enhanced recovery programmes mandate early mobilisation after surgery. In this study, patients were allowed to ambulate freely as part of their usual postoperative care, which may have produced some motion artefact on the continuous monitoring data; this may explain why the findings from this study show worse correlation when compared to previous studies which compared two stationary measurements. The patch algorithms are designed to identify and reject physiological signals corrupted by significant sources of noise inherent to the ambulatory nature of wireless monitoring; however, it is possible that respiratory rate data may have shown artefact from speech.

The findings must be interpreted within the limitations of the study. There were a relatively small number of patients in the study. Data completeness from the vital sign patch was low, especially for respiratory rate, although results for heart rate and temperature were similar to previous work (14). The reference standard, whilst clinically relevant, is inherently flawed. Early warning scores such as

For peer review only - http://bmjopen.bmj.com/site/about/guidelines.xhtml

NEWS are known to be limited by their user-dependent nature. Time and staffing pressures placed on nursing staff in an increasingly busy clinical environment may be driving the adoption of time-saving, less accurate techniques; in this study, heart rate was typically inferred from the pulse rate measured by a pulse oximeter, despite the fact that this is known to be less accurate than manual palpation of the radial pulse. In addition, manually-collected vital signs can be subject to the effects of 'white-coat hypertension'; heart rate, respiratory rate and temperature can be elevated simply by the arousal effect of the nurse interaction (20).

Deficits in the manually-recorded observations were particularly evident in the analysis of respiratory rate. Analysis of the manually-recorded values alone revealed a statistically unlikely preponderance of 18 breaths per minute, with a secondary peak at 16 breaths per minute. These peaks were not visible for the vital sign patch, suggesting that this is a measurement artefact in the way that manual measurements are made, rather than a real effect. It has been well described that respiratory rate is often miscalculated or omitted when calculated early warning scores (21,22). It is also recognised that clinical staff detect patient status in advance of manual measurements for an early warning score system 'by using information not currently encoded within it.'

The patch data for respiratory rate is also unlikely to be reliable, as a significant proportion of measurements were between 5 and 10 breaths per minute. This proportion of low values is much greater than those described in previously derived distributions from larger populations (23). There are also rapid fluctuations in respiratory rate which are physiologically implausible and may have been affected by patient movement, speech or coughing.

The manually-recorded temperature measurements showed plausible distributions and are likely to be accurate. The high bias between the nurse-measured temperatures and the patch data can be explained by the difference in measurement techniques. The patch measures skin temperature which may not accurately reflect the tympanic temperature measured by the nursing staff. Skin temperature is highly dependent on environmental factors such as the ambient temperature, clothing and blankets.

The reliability of the continuous temperature measurement is, however, limited. The time series analysis shows evidence of regular patch disconnection, indicated by rapid drops in temperature followed by increases consistent with conductive heating, or warming back up. These warm-up periods render the raw signals unreliable, although this limitation may be overcome through better signal processing. For instance, Clifton et al. used Bayesian change point analysis to detect step changes in temperature across a large study population. A similar approach may be used to determine disconnection on an individual patient basis(24).

Conclusions

The differences between manual and vital sign patch measurements for all three measured vital signs were outside of acceptable limits. On some occasions, this may be due to artefact in the continuous signal; this could be overcome through better signal processing. Other discrepancies may be due to errors during manual measurement. Whilst acknowledging the time pressures placed on nursing staff, inaccuracies in the manually-recorded data present an opportunity to increase awareness about the importance of manual observations, particularly with regard to methods of manual heart rate and respiratory rate measurements.

For peer review only - http://bmjopen.bmj.com/site/about/guidelines.xhtml

For peer review only

For peer review only - http://bmjopen.bmj.com/site/about/guidelines.xhtml

Figure legends

Figure 1: The SensiumVitals® monitoring patch. Image reproduced with permission from Sensium, Abingdon, UK.

Figure 2: Vital signs data for a single participant. The grey lines show the minute-by-minute vital sign values from the SensiumVitals® patch. The black markers show the median value of the SensiumVitals® vital signs (evaluated from +-10 mins of the nurse observation time). The red markers show the manually-recorded vital signs. Where there is a wide difference between the red and black markers at a single time point, this indicates disagreement between the two vital signs measurement techniques.

Figure 3: Scatter plot and marginal histogram of paired manual and SensiumVitals® heart rate observations; bpm=beats per minute.

Figure 4: Bland-Altman plot for heart rate with limits of agreement adjusted for repeated measures.

Figure 5: Scatter plot and marginal histogram of paired manual and SensiumVitals® respiratory rate observations; rpm=respirations per minute.

Figure 6: Bland-Altman plot for respiratory rate with limits of agreement adjusted for repeated measures.

Figure 7: Scatter plot and marginal histogram of paired manual and SensiumVitals® temperature observations.

Figure 8: Bland-Altman plot for temperature with limits of agreement adjusted for repeated measures.

For peer review only

For peer review only - http://bmjopen.bmj.com/site/about/guidelines.xhtml

Funding statement

Candice Downey is in possession of a Doctoral Research Fellowship (DRF-2016-09-037) supported by the National Institute for Health Research. DGJ received funding support through an NIHR Research Professorship. The research is supported by the NIHR infrastructure at Leeds.

The views expressed in this publication are those of the authors and not necessarily those of the NHS, the National Institute for Health Research, Health Education England or the Department of Health.

Competing interests

There are no known conflicts of interest associated with this work and there has been no significant financial support for this work that could have influenced its outcome.

Author contributions

CD, SN and DW were involved in the conception of the work. CD designed the study. DW provided methodological expertise. SN undertook the data collection. DW and CD performed the analysis and interpretation. CD and DW drafted the article. CD, SN, DW and DJ were involved in critical revision of the article and have given final approval of the version to be submitted.

Acknowledgements

The authors gratefully acknowledge the assistance of the Leeds Teaching Hospitals NHS Trust Surgical Research Team, with particular thanks to Mrs. Pauline Walton.

Data sharing statement

Research materials related to this work can be accessed on request from the corresponding author.

For peer review only

For peer review only - http://bmjopen.bmj.com/site/about/guidelines.xhtml

References

1. Downey CL, Tahir W, Randell R, Brown JM, Jayne DG. Strengths and limitations of early warning scores: A systematic review and narrative synthesis. Vol. 76, International Journal of Nursing Studies. 2017. p. 106–19.

2. Cardona-Morrell M, Prgomet M, Turner RM, Nicholson M, Hillman K. Effectiveness of continuous or intermittent vital signs monitoring in preventing adverse events on general wards: a systematic review and meta-analysis. Vol. 70, International Journal of Clinical Practice. 2016. p. 806–24.

3. DeVita MA, Smith GB, Adam SK, Adams-Pizarro I, Buist M, Bellomo R, et al. Identifying the hospitalised patient in crisis. A consensus conference on the afferent limb of Rapid Response Systems. Resuscitation. 2010;81(4):375–82.

4. Bonnici T, Tarassenko L, Clifton D, Watkinson P. The digital patient. Clin Med (Northfield Il). 2013;13(3):252–7.

5. Michard F. A sneak peek into digital innovations and wearable sensors for cardiac monitoring. J Clin Monit Comput. 2017 Apr;31(2):253–9.

6. Hofmann B, Welch H. New diagnostic tests: more harm than good. Br Med J. 2017;358:j3314.

7. Downey C, Randell R, Brown J, Jayne DG. Continuous Versus Intermittent Vital Signs Monitoring Using a Wearable, Wireless Patch in Patients Admitted to Surgical Wards: Pilot Cluster Randomized Controlled Trial. J Med Internet Res [Internet]. 2018 Dec 11;20(12):e10802. Available from: https://www.jmir.org/2018/12/e10802/

8. Downey CL, Brown JM, Jayne DG, Randell R. Patient attitudes towards remote continuous vital signs monitoring on general surgery wards: An interview study. Int J Med Inform. 2018;114.

9. Hernandez-Silveira M, Wieczorkowski-Rettinger K, Ang S, Burdett A. Preliminary assessment of the SensiumVitals®: A low-cost wireless solution for patient surveillance in the general wards. Proc Annu Int Conf IEEE Eng Med Biol Soc EMBS. 2015;2015–Novem:4931–7.

10. Backed I, Evidence BY. Sensium Whitepaper - The Deteriorating Patient. :1–5.

11. Hernandez-Silveira M, Ahmed K, Ang S-S, Zandari F, Mehta T, Weir R, et al. Assessment of the feasibility of an ultra-low power, wireless digital patch for the continuous ambulatory monitoring of vital signs. BMJ Open. 2015;5(5).

12. Downey CL, Croft J, Buckley H, Randell R, Brown JM, Jayne DG. Trial of Remote Continuous versus Intermittent NEWS monitoring after major surgery (TRaCINg): protocol for a feasibility randomised controlled trial. Pilot Feasibility Stud. 2018;4(1):112.

13. Royal College of Physicians. National Early Warning Score (NEWS) 2: Standardising the assessment of acute-illness severity in the NHS. Updated report of a working party. 2017.

14. Breteler MJM, Huizinga E, van Loon K, Leenen LPH, Dohmen DAJ, Kalkman CJ, et al. Reliability of wireless monitoring using a wearable patch sensor in high-risk surgical patients at a step-down unit in the Netherlands: a clinical validation study. BMJ Open [Internet]. 2018 Feb 1;8(2). Available from: http://bmjopen.bmj.com/content/8/2/e020162.abstract

15. Niven D, Gaudet J, Laupland K, Mrklas K, Roberts D, Stelfox H. Accuracy of peripheral

For peer review only - http://bmjopen.bmj.com/site/about/guidelines.xhtml

thermometers for estimating temperature: A systematic review and meta-analysis. Ann Intern Med [Internet]. 2015 Nov 17;163(10):768–77. Available from: http://dx.doi.org/10.7326/M15-1150

16. Carstensen B, Simpson J, Gurrin LC. Statistical models for assessing agreement in method comparison studies with replicate measurements. Int J Biostat. 2008;4(1).

17. Carstensen B, Gurrin L, Ekstrom C, Figurski M. MethComp: functions for analysis of agreement in method comparison studies. 2015. p. R package version 1.22.2.

18. R Core Team. R: A language and environment Statistical, statistical computing. 2018. p. R Foundation Computing, Vienna, Austria.

19. The International Surgical Outcomes Study Group. Global patient outcomes after elective surgery: prospective cohort study in 27 low-, middle-, and high-income countries. Br J Anaesth. 2016;117:601–9.

20. Franklin SS, Thijs L, Hansen TW, O'brien E, Staessen JA. White-coat hypertension: new insights from recent studies. Hypertension. 2013;62(6):982–7.

21. Nwulu U, Westwood D, Edwards D, Kelliher F, Coleman J. Adoption of an electronic observation chart with an integrated early warning scoring system on pilot wards: a descriptive report. Comput Informatics, Nurs. 2012;30(7):371–9.

22. Ludikhuize J, Smorenburg S, de Rooij S, de Jonge E. Identification of deteriorating patients on general wards; measurement of vital parameters and potential effectiveness of the Modified Early Warning Score. J Crit Care. 2012;27(4):424.e7-13.

23. Tarassenko L, Clifton DA, Pinsky MR, Hravnak MT, Woods JR, Watkinson PJ. Centile-based early warning scores derived from statistical distributions of vital signs. Resuscitation [Internet]. 2011;82(8):1013–8. Available from: http://www.sciencedirect.com/science/article/pii/S030095721100195X

24. Clifton DA, Wong D, Clifton L, Wilson S, Way R, Pullinger R, et al. A Large-Scale Clinical Validation of an Integrated Monitoring System in the Emergency Department. IEEE J Biomed Heal Informatics. 2013;17(4):835–42.

For peer review only

For peer review only - http://bmjopen.bmj.com/site/about/guidelines.xhtml

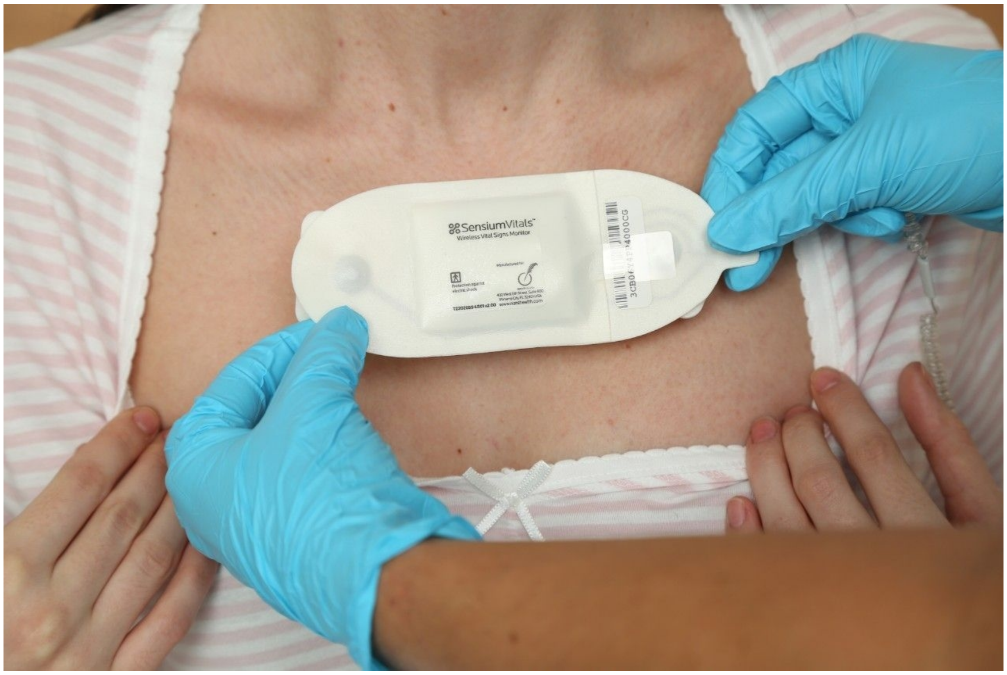

Figure 1: The SensiumVitals® monitoring patch. Image reproduced with permission from Sensium, Abingdon, UK.

451x300mm (72 x 72 DPI)

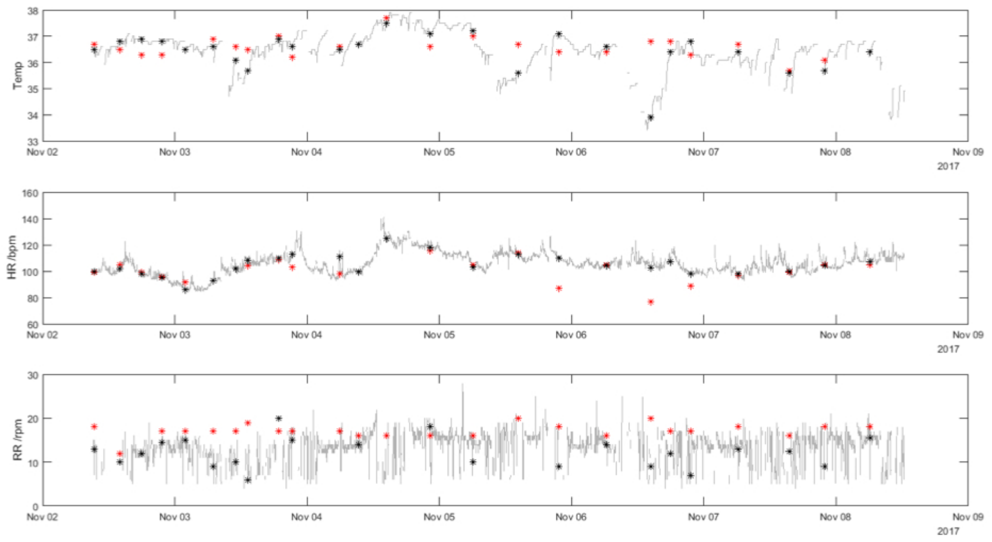

Figure 2: Vital signs data for a single participant. The grey lines show the minute-by-minute vital sign values from the SensiumVitals® patch. The black markers show the median value of the SensiumVitals® vital signs (evaluated from +-10 mins of the nurse observation time). The red markers show the manually-recorded vital signs. Where there is a wide difference between the red and black markers at a single time point, this indicates disagreement between the two vital signs measurement techniques.

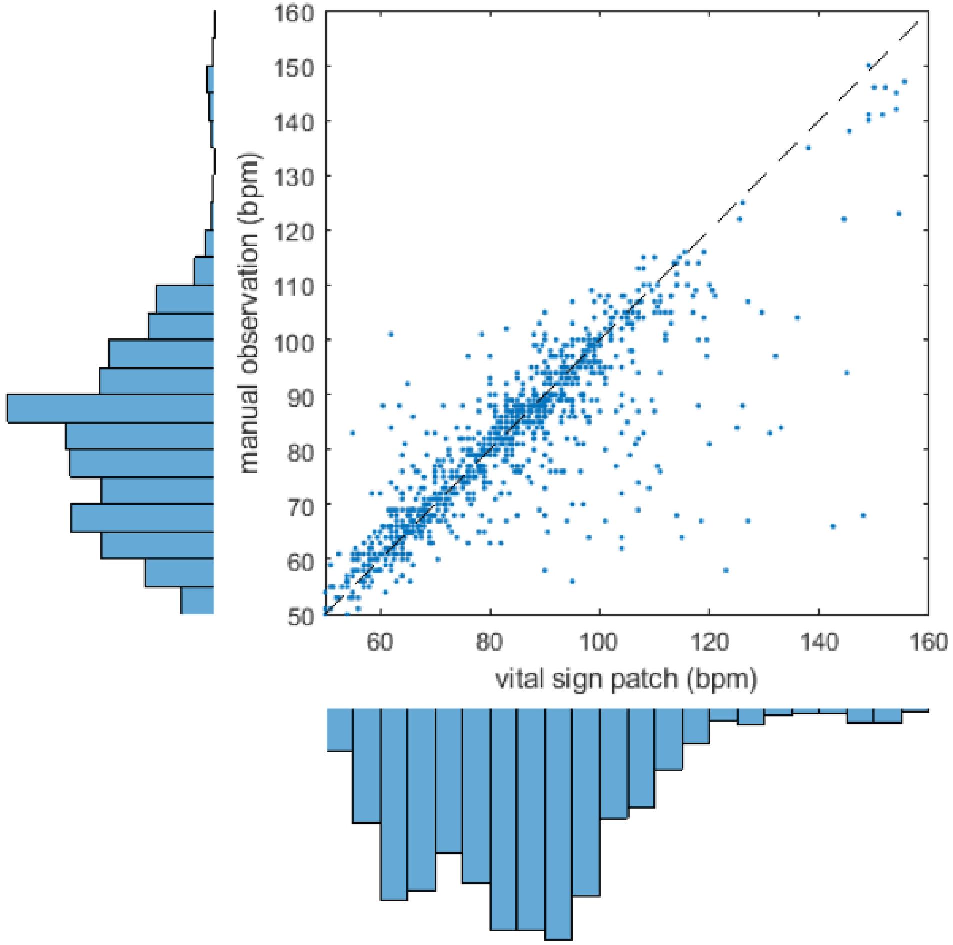

Figure 3: Scatter plot and marginal histogram of paired manual and SensiumVitals® heart rate observations; bpm=beats per minute.

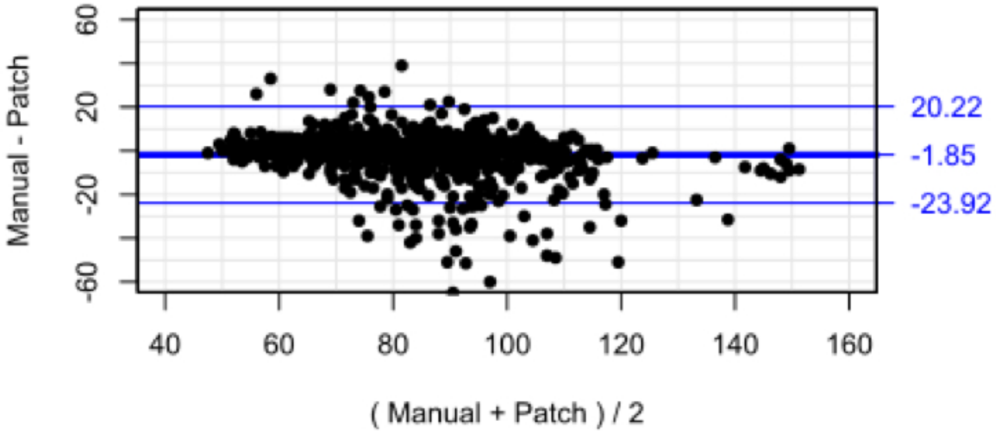

Figure 4: Bland-Altman plot for heart rate with limits of agreement adjusted for repeated measures.

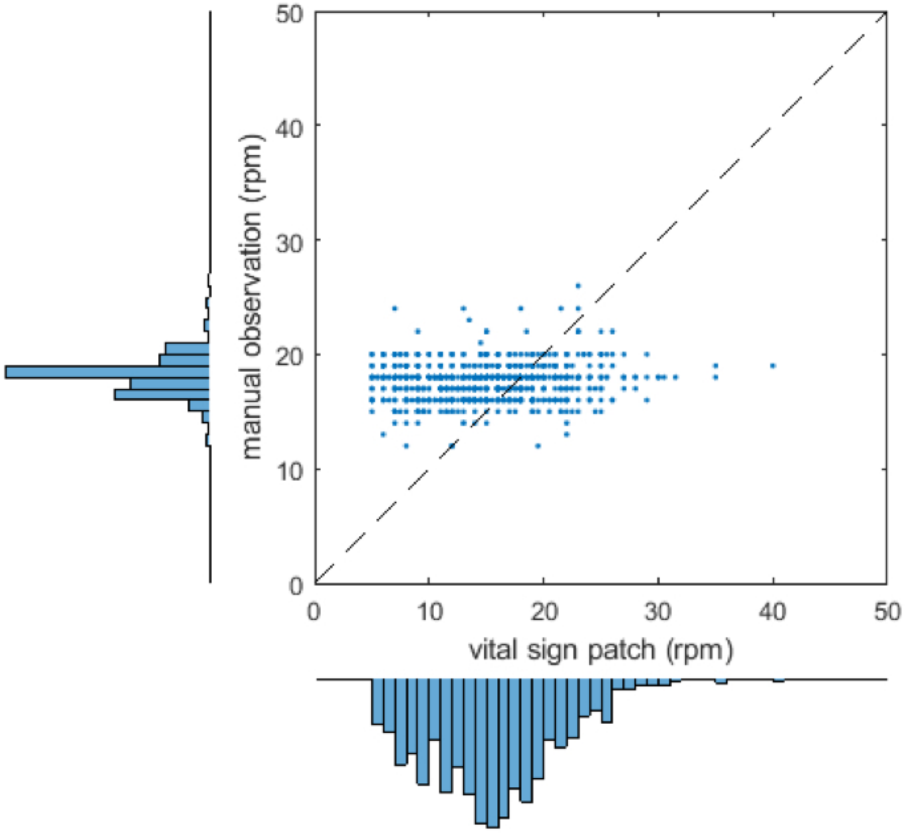

Figure 5: Scatter plot and marginal histogram of paired manual and SensiumVitals® respiratory rate observations; rpm=respirations per minute.

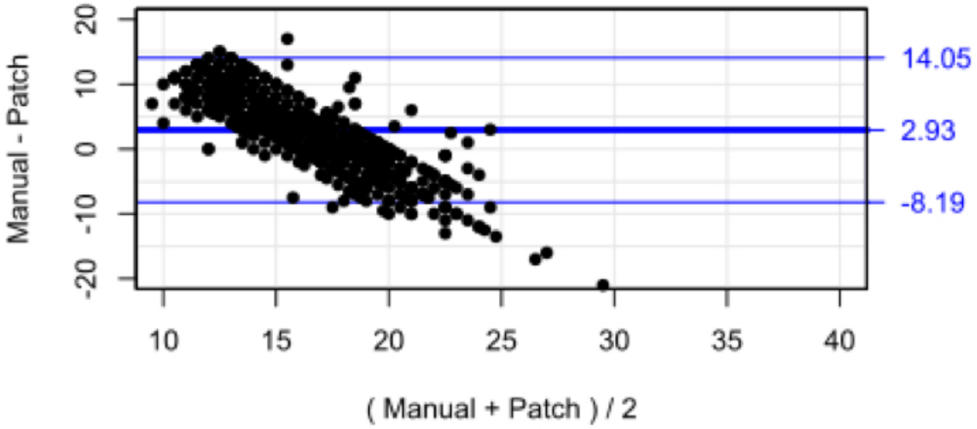

Figure 6: Bland-Altman plot for respiratory rate with limits of agreement adjusted for repeated measures.

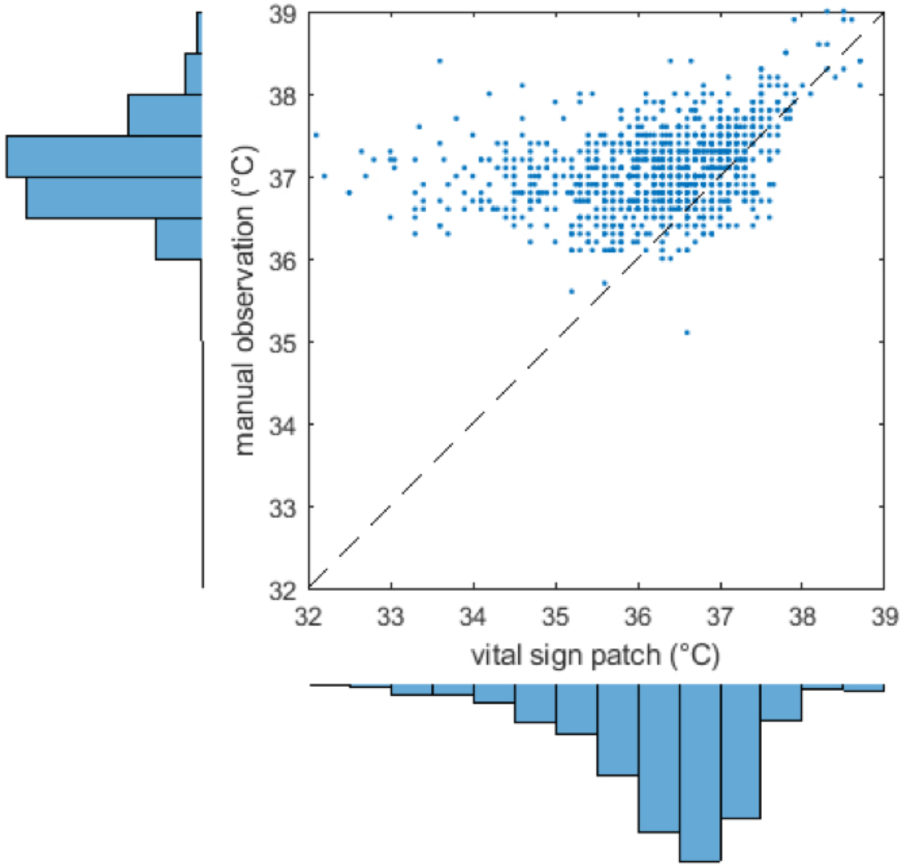

Figure 7: Scatter plot and marginal histogram of paired manual and SensiumVitals® temperature observations.

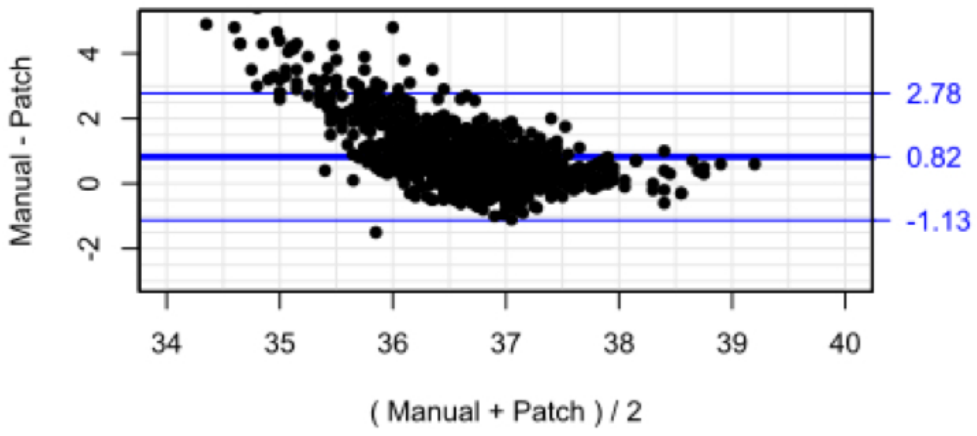

Figure 8: Bland-Altman plot for temperature with limits of agreement adjusted for repeated measures.

Supplementary Materials

Sensitivity analysis

Window size	HR (n = 1135)	RR (n = 1134)	Temp (n = 1132)
±2 mins	306	630	212
±5 mins	249	392	174
±10 mins	232	286	147

Missing pairs of data (i.e. no data within n minute window of nurse observation)

Window size	HR (bias, LoA)	RR (bias, LoA)	Temp (bias, LoA)
±2 mins	- 2.74 (-25.39, 19.91)	3.07 (-9.05 to 15.20)	0.82 (-1.21 to 2.86)
±5 mins	-2.35 (-24.68 to 19.98)	3.13 (-8.64 to 14.90)	0.82 (-1.23 to 2.87)
±10 mins	- 1.85 (-23.92 to 20.22)	2.93 (-8.19 to 14.05)	0.82 (-1.13 to 2.78)

Bland-Altman bias and 95% limits of agreement for ±2, ±5, ±10 window lengths of continuous vital sign patch data

For peer review only

BMJ Open

Reliability of a wearable wireless patch for continuous remote monitoring of vital signs in patients recovering from major surgery: a clinical validation study from the TRaCINg trial

Journal:	*BMJ Open*
Manuscript ID	bmjopen-2019-031150.R2
Article Type:	Research
Date Submitted by the Author:	12-Jul-2019

Complete List of Authors:	Downey, Candice ; University of Leeds, Leeds Institute of Medical Research at St. James's<br>Ng, Shu; University of Leeds, School of Medicine<br>Jayne, David; University of Leeds, Leeds Institute of Medical Research at St. James's<br>Wong, David; University of Manchester, Centre for Health Informatics

**Primary Subject Heading**:	Surgery
Secondary Subject Heading:	Evidence based practice, Health informatics
Keywords:	Vital Signs, continuous, monitoring, validation

SCHOLARONE™ Manuscripts

Reliability of a wearable wireless patch for continuous remote monitoring of vital signs in patients recovering from major surgery: a clinical validation study from the TRaCINg trial

CL Downey[1], S Ng[2], DG Jayne[1], DWong[3]

1. Leeds Institute of Medicine at St James's, Clinical Sciences Building,

St. James's University Hospital, University of Leeds, Leeds, LS9 7TF

2. School of Medicine, Worsley Building, University of Leeds, LS2 9JT
3. Centre for Health Informatics, University of Manchester, Manchester, M13 9GB

Correspondence to:

Miss Candice L Downey

Level 7, Clinical Sciences Building

St James's University Hospital

Leeds

LS9 7TF

Email: c.l.downey@leeds.ac.uk

Tel: 0113 2065281

For peer review only

Originality: This article is an original work, has not been published before, and is not being considered for publication elsewhere in its final form, in either printed or electronic media. It is not based on any previous communication to a society or meeting.

Keywords: Remote monitoring, vital signs, continuous, reliability, validation

For peer review only - http://bmjopen.bmj.com/site/about/guidelines.xhtml

For peer review only

For peer review only - http://bmjopen.bmj.com/site/about/guidelines.xhtml

Abstract

Objective

To validate whether a wearable remote vital signs monitor could accurately measure heart rate, respiratory rate and temperature in a post-surgical patient population at high risk of complications.

Design

Manually-recorded vital signs data were paired with vital signs data derived from the remote monitor set in patients participating in the TRaCINg study: a trial of continuous remote vital signs monitoring.

Setting

St James's University Hospital, United Kingdom.

Participants

51 patients who had undergone major elective general surgery.

Interventions

The intervention was the SensiumVitals® monitoring system. This is a wireless patch worn on the patient's chest that measures heart rate, respiratory rate and temperature continuously. The reference standard was nurse-measured manually-recorded vital signs.

Primary and secondary outcome measures

The primary outcomes were the 95% limits of agreement between manually-recorded and wearable patch vital sign recordings of heart rate, respiratory rate and temperature. The secondary outcomes were the percentage completeness of vital sign patch data for each vital sign.

Results

1,135 nurse observations were available for analysis. There was no clinically meaningful bias in heart rate (1.85 bpm), but precision was poor (95% limits of agreement -23.92 to 20.22 bpm). Agreement was poor for respiratory rate (bias 2.93 breaths per minute, 95% limits of agreement -8.19 to 14.05 breaths per minute) and temperature (bias 0.82°C, 95% limits of agreement -1.13°C to 2.78°C). Vital sign patch data completeness was 72.8% for temperature, 59.2% for heart rate and 34.1% for respiratory rate. Distributions of respiratory rate in manually-recorded measurements were clinically implausible.

Conclusions

The continuous monitoring system did not reliably provide heart rate consistent with nurse measurements. The accuracy of respiratory rate and temperature was outside of acceptable limits.

For peer review only - http://bmjopen.bmj.com/site/about/guidelines.xhtml

Limitations of the system could potentially be overcome through better signal processing. Whilst acknowledging the time pressures placed on nursing staff, inaccuracies in the manually-recorded data present an opportunity to increase awareness about the importance of manual observations, particularly with regard to methods of manual heart rate and respiratory rate measurements.

Article Summary: Strengths and limitations of this study

- Surgical patients are a population likely to benefit from continuous physiological monitoring.
- A large number of paired data sets were available for comparison.
- The reference standard is a clinically relevant comparison, and is standard of care throughout the UK.
- The accuracy of the reference standard is user-dependent.

For peer review only

For peer review only - http://bmjopen.bmj.com/site/about/guidelines.xhtml

1. Introduction

Physiological monitoring using early warning score systems is effective but limited by its intermittent nature (1). It is hypothesised that continuous vital signs monitoring may allow earlier detection of patient deterioration and thereby improve patient outcomes, but existing evidence is limited (2). A consensus of international experts in safety and healthcare technology concluded that, if technically possible and affordable, all patients who are for active treatment should be continuously monitored (3).

Until recently, continuous vital signs monitoring was limited to critical care areas because it required high staff-to-patient ratios and cumbersome equipment which tethered the patient to the bed-space, thereby inhibiting patient mobility and recovery. When hard-wired monitoring was implemented on a general ward, only 16% of patients remained connected in a 72-hour period (4).

New remote monitoring devices, consisting of wearable sensors and aided by wireless data transmission, allow the patient to ambulate freely whilst enjoying the presumed advantages of extra monitoring. Since 2002, a number of such tools have received the United States Food and Drug Administration (FDA) clearance, indicating that they are safe and effective, but clinical studies are required to demonstrate their utility in the inpatient setting (5,6).

A remote monitoring device with a considerable amount of clinical evidence is the SensiumVitals® patch (Figure 1)(7–10). Attached to the patient's chest with two ECG electrodes, the device monitors heart rate, respiratory rate and skin temperature continuously. The data are transmitted wirelessly every two minutes to a central monitoring station or a mobile device carried by the patient's nurse. This alerts the healthcare worker when there is deviation from pre-set physiological norms, alerting staff to potential patient deterioration.

The patch records respiratory rate by means of impedance pneumography and heart rate through single-lead ECG activity. Temperature is measured by a temperature-sensitive resistor. Once a physiological signal is fully acquired, it is processed by its associated embedded algorithm running inside the in-built processing unit, which enables the transmission of the resultant values to a nearby intranet hot-spot for onward transmission to the central monitoring system.

The underlying technology incorporated into such devices is well understood, but there is limited evidence for its reliability in the clinical setting. One previous study exists which validated the accuracy of the SensiumVitals® system in 61 hospital patients. The patients were monitored at rest for a maximum of two hours, and the device was tested against a conventional bedside clinical monitor using capnographic respiratory rate (11). This does not reflect the true clinical environment, which challenges such devices to provide monitoring continuity over several days in ambulatory patients.

In this study, we validated the accuracy of the SensiumVitals® system to measure heart rate, respiratory rate and temperature in a post-surgical patient population at high risk for complications. The reference standard were manually-recorded vital signs as part of the National Early Warning Score (NEWS). The objective of this study was to assess whether the wireless patch system is able

For peer review only - http://bmjopen.bmj.com/site/about/guidelines.xhtml

to reliably measure vital signs continuously in the clinical setting, and to determine how well it compares to manually-recorded measurements.

2. Methods

Ethical approval was granted on 10th October 2017 by the Yorkshire & The Humber – Leeds West Research Ethics Committee, ref: 17/YH/0180. Informed consent to participate was obtained from all participants in the study.

2.1 Study design

All participants were enrolled in the TRaCINg study, the protocol for which has been published previously (12). This was a single-centre, feasibility, randomised, controlled, parallel group trial of continuous remote vital signs monitoring for patients who had undergone major elective general surgery at St James's University Hospital, Leeds, United Kingdom. Participants were individually randomised on a 1:1 basis to receive either remote monitoring plus NEWS or monitoring by NEWS alone. This paper describes the data from participants randomised to the remote monitoring arm, who wore the SensiumVitals® patch during their hospital admission. The TRaCINg study is listed on the ISRCTN registry with study ID ISRCTN16601772 (http://www.isrctn.com/ISRCTN16601772).

2.2 Patient and public involvement

Patients and the public were involved in the design of the randomised controlled trial, but were not involved in the design of this validation study.

2.3 Data collection

Vital signs data was collected for each participant from two sources. The SensiumVitals® vital sign data were documented at 2-minute intervals and collected from a hospital desktop computer using data-acquisition software developed by Sensium. These data had been pre-processed to discard signals that were subject to gross electrical or motion artefact (11). Patients were allowed to ambulate whilst wearing the monitoring patch; however, due to the major surgery they had undergone, most patients remained at their bedsides for the duration of their hospital stay.

NEWS data were collected at regular intervals, depending on the patients' status and based on the NEWS protocol(13). Typically, vital signs were collected at the bedside, with the patients either sitting or lying down, by members of the nursing staff who were blinded to the SensiumVitals® vital sign data: pulse rate was measured using the pulse oximeter on a multi-parameter portable vital signs monitor; temperature was measured using a tympanic thermometer; respiratory rate was measured manually. The NEWS scores and their component parts were documented electronically. Researchers collected manually-recorded heart rate, respiratory rate and temperature data from the hospital's electronic patient record. Other vital signs collected by the nursing staff as part of the early warning score, such as oxygen saturations, were not extracted.

2.4 Data processing

The two data sources were linked using NHS number and timestamp and consolidated into a single deidentified spreadsheet. Paired data to a NEWS observation was derived from the SensiumVitals®

For peer review only - http://bmjopen.bmj.com/site/about/guidelines.xhtml

continuous data set by using the median vital sign value within a ±10-minute window of a manually-recorded observation. The time window was used to account for differences between the nurses' manually-documented times and the automatic timestamps from the vital sign patch. The median value within this window was used to eliminate the impact of intermittent sensor noise.

2.5 Outcomes

The primary outcomes were the 95% limits of agreement between manual nurse observations and wearable vital sign patch recordings of Heart Rate (HR), Respiratory Rate (RR) and Temperature (Temp). Following precedent, we defined clinical acceptability to be *max* ±10% for HR and RR (or ±3 breaths per minute or ±5 beats per minute) and 0.5°C for Temp (14,15). The secondary outcome was the average percentage completeness of continuous patch data.

2.6 Statistical Analysis

For each vital sign, we first visually inspected the paired vital sign measurements via scatter plots, in addition to the raw time series vital signs from the Sensium patch.

Measurements were then formally compared using Bland-Altman analysis. In this analysis, the mean difference between the SensiumVitals® data and the nurse observations and the 95% Limits of Agreement are calculated. We adjusted for multiple measurements from the same subject using a model in which time of measurement is modelled as a random effect(16). This avoids bias caused by differences in number of measurements per patient. We also reported the Pearson correlation coefficient and the root mean squared (RMS) error for each vital sign.

In secondary analysis, we first assessed the average percentage completeness of the continuous patch data per patient. The numerator was defined as the number of two-minute periods in which vital sign data were provided by the patch. The denominator was the number of 2-minute periods that span the time during which the patch was transmitting data. These time points were preferred to admission and discharge from ward times because the patch may not have been worn for the patient's entire ward admission. In sensitivity analyses, we repeated both the Bland-Altman analyses using ±2 and ±2 minute windows of continuous data.

Analyses were undertaken using MATLAB R2017b (The MathWorks Inc., Massachusetts, USA) and the R Methcomp package (17,18).

3. Results

Fifty one patients were recruited to the intervention arm of the TRaCINg study between October 2017 and April 2018. The median number of manually-recorded observation sets was 19 per patient (range 2 to 73 sets of vital signs measurements). There were 1,135 nurse observations available for analysis. All observations had a documented heart rate. Four observations had missing observations, 1 for respiratory rate and 3 for temperature. Vital sign traces for one participant over the course of their entire hospital stay are shown in Figure 2.

For peer review only - http://bmjopen.bmj.com/site/about/guidelines.xhtml

3.1 Heart Rate

Figure 3 shows the scatterplot of nurse-recorded heart rate against the SensiumVitals® patch. There is reasonable correlation between the two measurements ($R^2 = 0.67$, $p < 0.001$). The mean and (standard deviation) for manual and wearable heart rates are 81.6 (16.2) beats per minute (bpm) and 84.3 (19.3) bpm, respectively. The mean percentage completeness of continuous patch data for heart rate was 59.2%. In addition, visual inspection of the example vital sign traces show good agreement between the measurements. The Bland-Altman bias (Figure 4) was 1.85 bpm, with 95% limits of agreement -23.92 to 20.22 bpm. The RMS error was 11.25 bpm. The limits of agreement and RMS error exceeded the acceptability criterion.

3.2 Respiratory Rate

Figure 5 shows the scatterplot of nurse-recorded respiratory rate against the SensiumVitals® patch data. There is no correlation between the two measurements methods ($R^2 = 0.01$, $p < 0.001$). The mean and standard deviation for manual and wearable respiratory rate were 17.6 (1.58) breaths per minute and 15.0 (5.5) breaths per minute, respectively. The mean percentage completeness of continuous patch data for respiratory rate data was 31.4%.

Visual inspection of the histogram for manually-recorded respiratory rate shows a large peak at 18 breaths per minute, and a secondary peak at 16 breaths per minute. This result is unexpected for a natural physiological parameter, which may be expected to vary smoothly over the full range of values. Indeed, the peaks do not appear on the vital sign patch histogram. Inspection of the vital sign patch histogram indicates a significant proportion of measurements between 5 and 10 breaths per minute. No manually-recorded respiratory rates were recorded in this range. The Bland-Altman bias (Figure 6) was 2.93 breaths per minute, with 95% limits of agreement -8.19 to 14.05 breaths per minute. The RMS error was 6.14 breaths per minute and the limits of agreement are wider than the pre-specified acceptable error of 3 breaths/min.

3.3 Temperature

Figure 7 is a scatterplot of temperatures recorded by nurses versus those measured by the SensiumVitals® patch. Histograms for each measurement method are presented alongside the x- and y- axes. There is low correlation between the two measurement methods ($R^2 = 0.13$, $p < 0.001$). The mean and (standard deviation) of manual temperature and wearable temperature were 37.1℃ (0.5℃) and 36.4℃ (1.0℃). Further inspection of the vital sign time series in Figures 1 and 2 shows multiple clinically implausible fluctuations of up to 2 °C within 2 hours within each time series. The mean percentage completeness of continuous patch data for temperature was 72.8%.

Initial visual inspection was therefore sufficient to show that the patch-derived temperature is not a suitable proxy for core temperature, as measured by tympanic thermometer. The Bland-Altman bias (Figure 8) was 0.82℃, with 95% limits of agreement -1.13℃ to 2.78℃. The RMS error was 1.28℃. In addition to large systematic bias between the two methods, the limits of agreement did not meet the pre-defined clinical acceptability criterion (0.5°C).

For peer review only - http://bmjopen.bmj.com/site/about/guidelines.xhtml

In a sensitivity analysis, all Bland-Altman analyses was repeated using ±2 and ±5 minute windows of vital sign patch data. There were no meaningful differences in the bias or limits of agreement [Supplementary Material].

4. Discussion

In this 51-patient validation study, temperature, respiratory rate, and heart rate measurements obtained from a wearable vital sign patch were compared with manually-recorded observations by nursing staff. Whilst there was reasonable correlation between the two methods for heart rate measurements, there were large discrepancies in many instances, as indicated by the Bland Altman analysis. It is not clear whether there were errors in the manual observation, in the vital sign patch, or both. There was low correlation for respiratory rate and temperature. The differences between manual and vital sign patch measurements for all three measured vital signs were outside of acceptable limits.

An advantage of the study design is the collection of a large number of data points for analysis. The approach is clinically valid, as the NEWS system is the national standard for vital signs monitoring in the United Kingdom. The surgical patient population is a clinically relevant cohort. There are high rates of complications after major surgery (19), but many surgical complications, such as sepsis, are attenuated by early detection. By virtue of their suitability for surgery, patients experiencing severe complications are likely to be candidates for full active management and escalation of care. They are therefore a population likely to benefit from reliable continuous physiological monitoring.

There are few clinical evaluations of continuous vital signs monitoring in the literature. Previous validation studies have studied participants who are confined to their bed space by wired monitoring equipment (11,14). In the surgical setting, enhanced recovery programmes mandate early mobilisation after surgery. In this study, patients were allowed to ambulate freely as part of their usual postoperative care, which may have produced some motion artefact on the continuous monitoring data; this may explain why the findings from this study show worse correlation when compared to previous studies which compared two stationary measurements. The patch algorithms are designed to identify and reject physiological signals corrupted by significant sources of noise inherent to the ambulatory nature of wireless monitoring; however, it is possible that respiratory rate data may have shown artefact from speech.

The findings must be interpreted within the limitations of the study. There were a relatively small number of patients in the study. Data completeness from the vital sign patch was low, especially for respiratory rate, although results for heart rate and temperature were similar to previous work (14). The reference standard, whilst clinically relevant, is inherently flawed. Early warning scores such as NEWS are known to be limited by their user-dependent nature. Time and staffing pressures placed on nursing staff in an increasingly busy clinical environment may be driving the adoption of time-saving, less accurate techniques; in this study, heart rate was typically inferred from the pulse rate measured by a pulse oximeter, despite the fact that this is known to be less accurate than manual palpation of the radial pulse. In addition, manually-collected vital signs can be subject to the effects

For peer review only - http://bmjopen.bmj.com/site/about/guidelines.xhtml

of 'white-coat hypertension'; heart rate, respiratory rate and temperature can be elevated simply by the arousal effect of the nurse interaction (20).

Deficits in the manually-recorded observations were particularly evident in the analysis of respiratory rate. Analysis of the manually-recorded values alone revealed a statistically unlikely preponderance of 18 breaths per minute, with a secondary peak at 16 breaths per minute. These peaks were not visible for the vital sign patch, suggesting that this is a measurement artefact in the way that manual measurements are made, rather than a real effect. It has been well described that respiratory rate is often miscalculated or omitted when calculated early warning scores (21,22). It is also recognised that clinical staff detect patient status in advance of manual measurements for an early warning score system 'by using information not currently encoded within it.'

The patch data for respiratory rate is also unlikely to be reliable, as a significant proportion of measurements were between 5 and 10 breaths per minute. This proportion of low values is much greater than those described in previously derived distributions from larger populations (23). There are also rapid fluctuations in respiratory rate which are physiologically implausible and may have been affected by patient movement, speech or coughing.

The manually-recorded temperature measurements showed plausible distributions and are likely to be accurate. The high bias between the nurse-measured temperatures and the patch data can be explained by the difference in measurement techniques. The patch measures skin temperature which may not accurately reflect the tympanic temperature measured by the nursing staff. Skin temperature is highly dependent on environmental factors such as the ambient temperature, clothing and blankets.

The reliability of the continuous temperature measurement is, however, limited. The time series analysis shows evidence of regular patch disconnection, indicated by rapid drops in temperature followed by increases consistent with conductive heating, or warming back up. These warm-up periods render the raw signals unreliable, although this limitation may be overcome through better signal processing. For instance, Clifton et al. used Bayesian change point analysis to detect step changes in temperature across a large study population. A similar approach may be used to determine disconnection on an individual patient basis(24).

For peer review only

Conclusions

The differences between manual and vital sign patch measurements for all three measured vital signs were outside of acceptable limits. On some occasions, this may be due to artefact in the continuous signal; this could be overcome through better signal processing. Other discrepancies may be due to errors during manual measurement. Whilst acknowledging the time pressures placed on nursing staff, inaccuracies in the manually-recorded data present an opportunity to increase awareness about the importance of manual observations, particularly with regard to methods of manual heart rate and respiratory rate measurements.

For peer review only - http://bmjopen.bmj.com/site/about/guidelines.xhtml

Figure legends

Figure 1: The SensiumVitals® monitoring patch. Image reproduced with permission from Sensium, Abingdon, UK.

Figure 2: Vital signs data for a single participant. The grey lines show the minute-by-minute vital sign values from the SensiumVitals® patch. The black markers show the median value of the SensiumVitals® vital signs (evaluated from +-10 mins of the nurse observation time). The red markers show the manually-recorded vital signs. Where there is a wide difference between the red and black markers at a single time point, this indicates disagreement between the two vital signs measurement techniques.

Figure 3: Scatter plot and marginal histogram of paired manual and SensiumVitals® heart rate observations; bpm=beats per minute.

Figure 4: Bland-Altman plot for heart rate with limits of agreement adjusted for repeated measures.

Figure 5: Scatter plot and marginal histogram of paired manual and SensiumVitals® respiratory rate observations; rpm=respirations per minute.

Figure 6: Bland-Altman plot for respiratory rate with limits of agreement adjusted for repeated measures.

Figure 7: Scatter plot and marginal histogram of paired manual and SensiumVitals® temperature observations.

Figure 8: Bland-Altman plot for temperature with limits of agreement adjusted for repeated measures.

For peer review only

Funding statement

Candice Downey is in possession of a Doctoral Research Fellowship (DRF-2016-09-037) supported by the National Institute for Health Research. DGJ received funding support through an NIHR Research Professorship. The research is supported by the NIHR infrastructure at Leeds.

The views expressed in this publication are those of the authors and not necessarily those of the NHS, the National Institute for Health Research, Health Education England or the Department of Health.

Competing interests

There are no known conflicts of interest associated with this work and there has been no significant financial support for this work that could have influenced its outcome.

Author contributions

CD, SN and DW were involved in the conception of the work. CD designed the study. DW provided methodological expertise. SN undertook the data collection. DW and CD performed the analysis and interpretation. CD and DW drafted the article. CD, SN, DW and DJ were involved in critical revision of the article and have given final approval of the version to be submitted.

Acknowledgements

The authors gratefully acknowledge the assistance of the Leeds Teaching Hospitals NHS Trust Surgical Research Team, with particular thanks to Mrs. Pauline Walton.

Data sharing statement

Research materials related to this work can be accessed on request from the corresponding author.

For peer review only

References

1. Downey CL, Tahir W, Randell R, Brown JM, Jayne DG. Strengths and limitations of early warning scores: A systematic review and narrative synthesis. Vol. 76, International Journal of Nursing Studies. 2017. p. 106–19.

2. Cardona-Morrell M, Prgomet M, Turner RM, Nicholson M, Hillman K. Effectiveness of continuous or intermittent vital signs monitoring in preventing adverse events on general wards: a systematic review and meta-analysis. Vol. 70, International Journal of Clinical Practice. 2016. p. 806–24.

3. DeVita MA, Smith GB, Adam SK, Adams-Pizarro I, Buist M, Bellomo R, et al. Identifying the hospitalised patient in crisis. A consensus conference on the afferent limb of Rapid Response Systems. Resuscitation. 2010;81(4):375–82.

4. Bonnici T, Tarassenko L, Clifton D, Watkinson P. The digital patient. Clin Med (Northfield Il). 2013;13(3):252–7.

5. Michard F. A sneak peek into digital innovations and wearable sensors for cardiac monitoring. J Clin Monit Comput. 2017 Apr;31(2):253–9.

6. Hofmann B, Welch H. New diagnostic tests: more harm than good. Br Med J. 2017;358:j3314.

7. Downey C, Randell R, Brown J, Jayne DG. Continuous Versus Intermittent Vital Signs Monitoring Using a Wearable, Wireless Patch in Patients Admitted to Surgical Wards: Pilot Cluster Randomized Controlled Trial. J Med Internet Res [Internet]. 2018 Dec 11;20(12):e10802. Available from: https://www.jmir.org/2018/12/e10802/

8. Downey CL, Brown JM, Jayne DG, Randell R. Patient attitudes towards remote continuous vital signs monitoring on general surgery wards: An interview study. Int J Med Inform. 2018;114.

9. Hernandez-Silveira M, Wieczorkowski-Rettinger K, Ang S, Burdett A. Preliminary assessment of the SensiumVitals®: A low-cost wireless solution for patient surveillance in the general wards. Proc Annu Int Conf IEEE Eng Med Biol Soc EMBS. 2015;2015–Novem:4931–7.

10. Backed I, Evidence BY. Sensium Whitepaper - The Deteriorating Patient. :1–5.

11. Hernandez-Silveira M, Ahmed K, Ang S-S, Zandari F, Mehta T, Weir R, et al. Assessment of the feasibility of an ultra-low power, wireless digital patch for the continuous ambulatory monitoring of vital signs. BMJ Open. 2015;5(5).

12. Downey CL, Croft J, Buckley H, Randell R, Brown JM, Jayne DG. Trial of Remote Continuous versus Intermittent NEWS monitoring after major surgery (TRaCINg): protocol for a feasibility randomised controlled trial. Pilot Feasibility Stud. 2018;4(1):112.

13. Royal College of Physicians. National Early Warning Score (NEWS) 2: Standardising the assessment of acute-illness severity in the NHS. Updated report of a working party. 2017.

14. Breteler MJM, Huizinga E, van Loon K, Leenen LPH, Dohmen DAJ, Kalkman CJ, et al. Reliability of wireless monitoring using a wearable patch sensor in high-risk surgical patients at a step-down unit in the Netherlands: a clinical validation study. BMJ Open [Internet]. 2018 Feb 1;8(2). Available from: http://bmjopen.bmj.com/content/8/2/e020162.abstract

15. Niven D, Gaudet J, Laupland K, Mrklas K, Roberts D, Stelfox H. Accuracy of peripheral

For peer review only - http://bmjopen.bmj.com/site/about/guidelines.xhtml

thermometers for estimating temperature: A systematic review and meta-analysis. Ann Intern Med [Internet]. 2015 Nov 17;163(10):768–77. Available from: http://dx.doi.org/10.7326/M15-1150

16. Carstensen B, Simpson J, Gurrin LC. Statistical models for assessing agreement in method comparison studies with replicate measurements. Int J Biostat. 2008;4(1).

17. Carstensen B, Gurrin L, Ekstrom C, Figurski M. MethComp: functions for analysis of agreement in method comparison studies. 2015. p. R package version 1.22.2.

18. R Core Team. R: A language and environment Statistical, statistical computing. 2018. p. R Foundation Computing, Vienna, Austria.

19. The International Surgical Outcomes Study Group. Global patient outcomes after elective surgery: prospective cohort study in 27 low-, middle-, and high-income countries. Br J Anaesth. 2016;117:601–9.

20. Franklin SS, Thijs L, Hansen TW, O'brien E, Staessen JA. White-coat hypertension: new insights from recent studies. Hypertension. 2013;62(6):982–7.

21. Nwulu U, Westwood D, Edwards D, Kelliher F, Coleman J. Adoption of an electronic observation chart with an integrated early warning scoring system on pilot wards: a descriptive report. Comput Informatics, Nurs. 2012;30(7):371–9.

22. Ludikhuize J, Smorenburg S, de Rooij S, de Jonge E. Identification of deteriorating patients on general wards; measurement of vital parameters and potential effectiveness of the Modified Early Warning Score. J Crit Care. 2012;27(4):424.e7-13.

23. Tarassenko L, Clifton DA, Pinsky MR, Hravnak MT, Woods JR, Watkinson PJ. Centile-based early warning scores derived from statistical distributions of vital signs. Resuscitation [Internet]. 2011;82(8):1013–8. Available from: http://www.sciencedirect.com/science/article/pii/S030095721100195X

24. Clifton DA, Wong D, Clifton L, Wilson S, Way R, Pullinger R, et al. A Large-Scale Clinical Validation of an Integrated Monitoring System in the Emergency Department. IEEE J Biomed Heal Informatics. 2013;17(4):835–42.

For peer review only

For peer review only - http://bmjopen.bmj.com/site/about/guidelines.xhtml

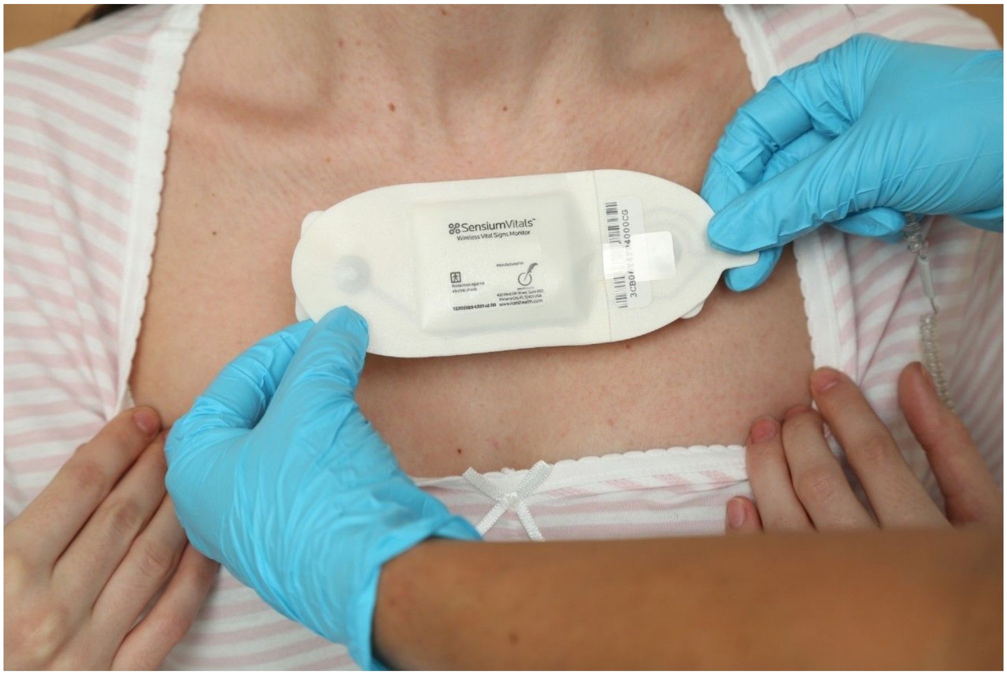

Figure 1: The SensiumVitals® monitoring patch. Image reproduced with permission from Sensium, Abingdon, UK.

451x300mm (72 x 72 DPI)

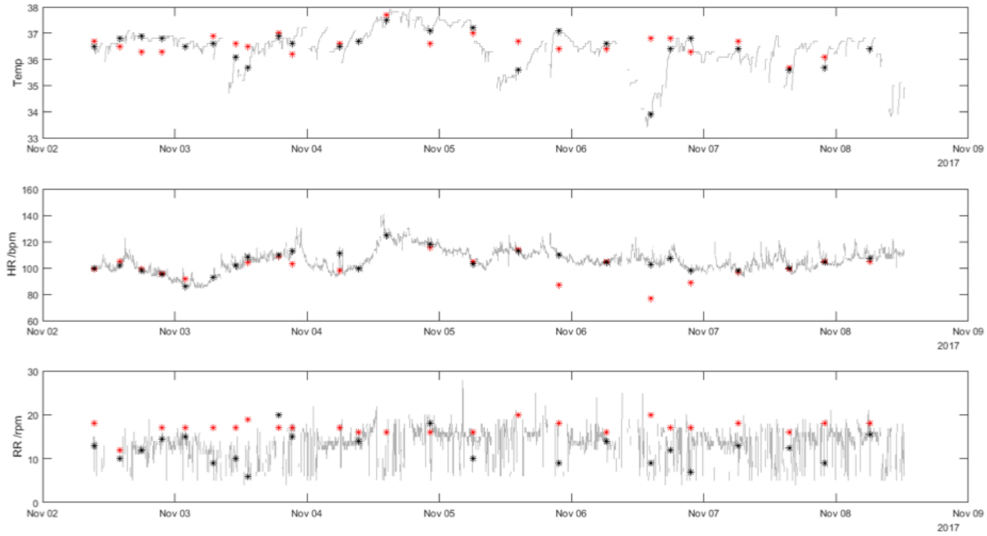

Figure 2: Vital signs data for a single participant. The grey lines show the minute-by-minute vital sign values from the SensiumVitals® patch. The black markers show the median value of the SensiumVitals® vital signs (evaluated from +-10 mins of the nurse observation time). The red markers show the manually-recorded vital signs. Where there is a wide difference between the red and black markers at a single time point, this indicates disagreement between the two vital signs measurement techniques.

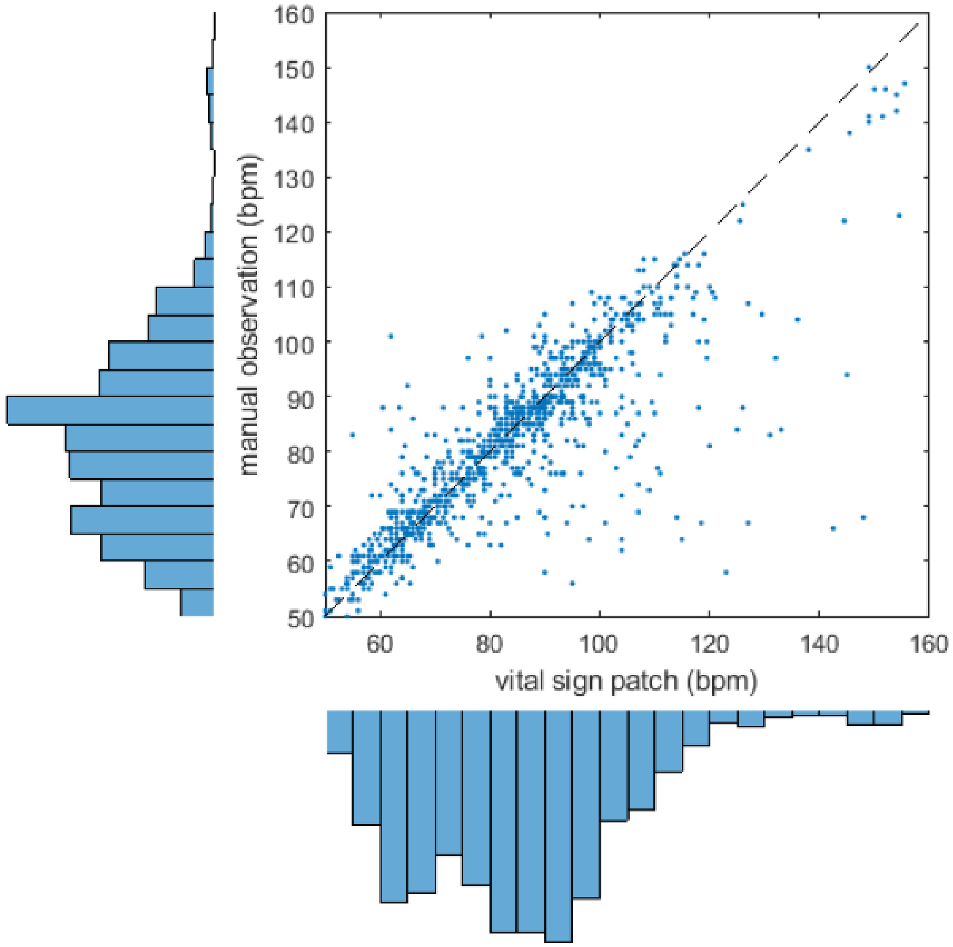

Figure 3: Scatter plot and marginal histogram of paired manual and SensiumVitals® heart rate observations; bpm=beats per minute.

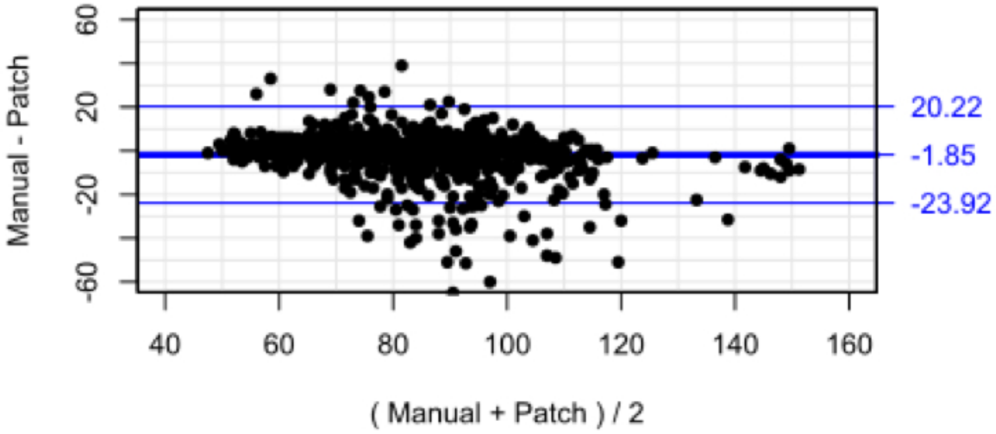

Figure 4: Bland-Altman plot for heart rate with limits of agreement adjusted for repeated measures.

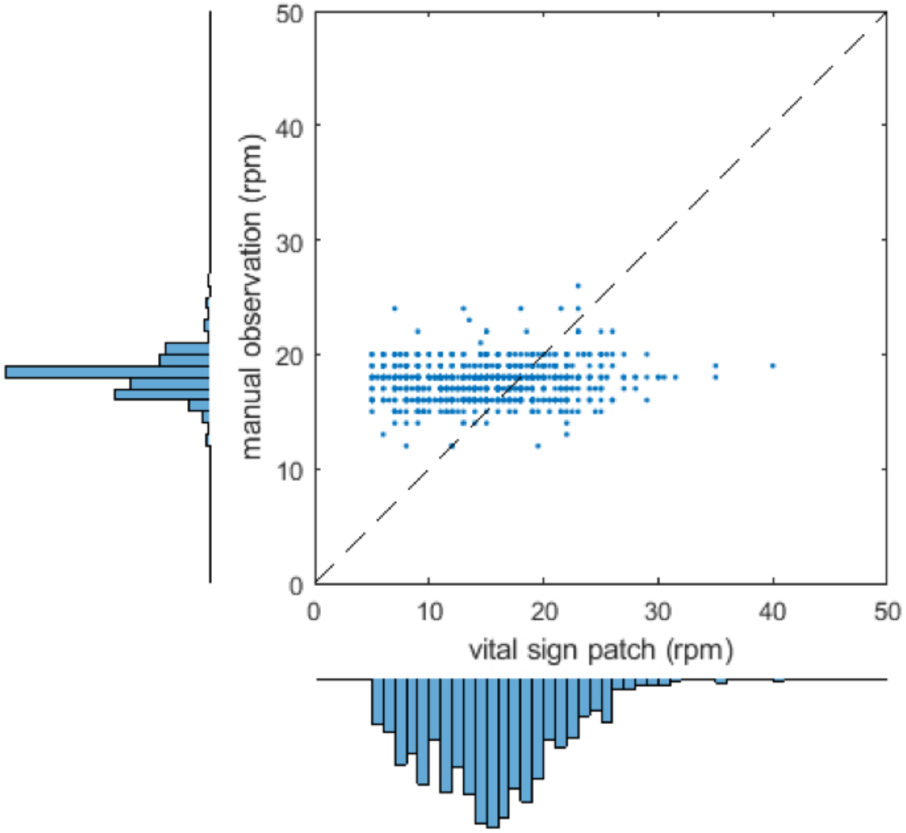

Figure 5: Scatter plot and marginal histogram of paired manual and SensiumVitals® respiratory rate observations; rpm=respirations per minute.

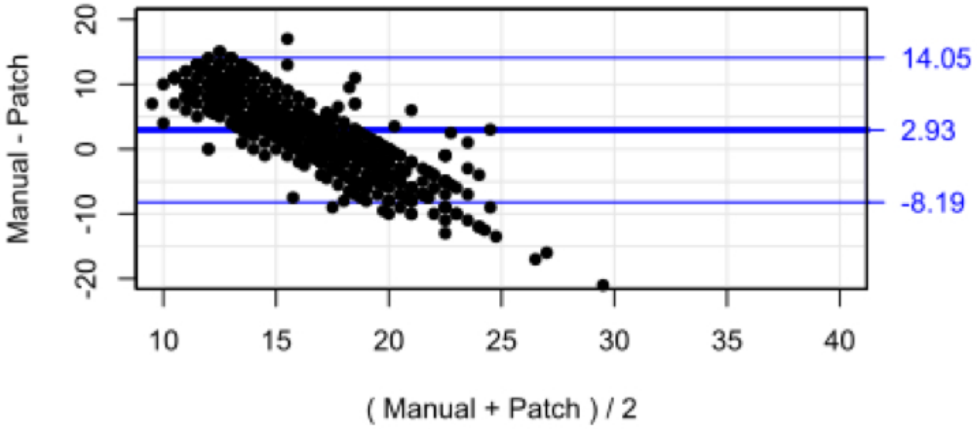

Figure 6: Bland-Altman plot for respiratory rate with limits of agreement adjusted for repeated measures.

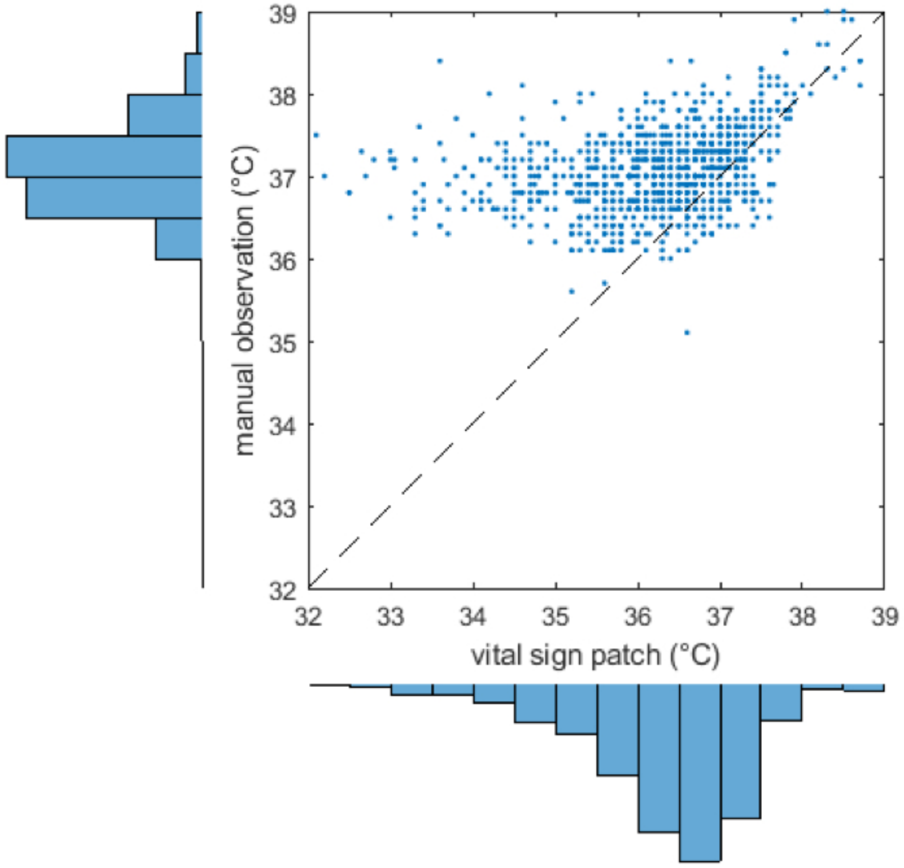

Figure 7: Scatter plot and marginal histogram of paired manual and SensiumVitals® temperature observations.

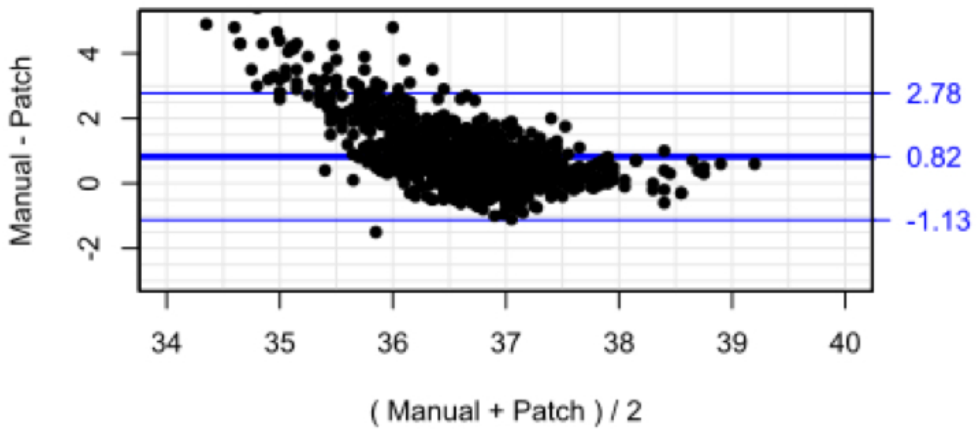

Figure 8: Bland-Altman plot for temperature with limits of agreement adjusted for repeated measures.

Supplementary Materials

Sensitivity analysis

Window size	HR (n = 1135)	RR (n = 1134)	Temp (n = 1132)
±2 mins	306	630	212
±5 mins	249	392	174
±10 mins	232	286	147

Missing pairs of data (i.e. no data within n minute window of nurse observation)

Window size	HR (bias, LoA)	RR (bias, LoA)	Temp (bias, LoA)
±2 mins	- 2.74 (-25.39, 19.91)	3.07 (-9.05 to 15.20)	0.82 (-1.21 to 2.86)
±5 mins	-2.35 (-24.68 to 19.98)	3.13 (-8.64 to 14.90)	0.82 (-1.23 to 2.87)
±10 mins	- 1.85 (-23.92 to 20.22)	2.93 (-8.19 to 14.05)	0.82 (-1.13 to 2.78)

Bland-Altman bias and 95% limits of agreement for ±2, ±5, ±10 window lengths of continuous vital sign patch data

For peer review only - http://bmjopen.bmj.com/site/about/guidelines.xhtml
